# Factors Influencing Time to Treatment Initiation for Breast Cancer in Ethiopia

**DOI:** 10.1002/cam4.71439

**Published:** 2025-12-04

**Authors:** Anteneh Ayelign Kibret, Jason Jiang, Edom Seife Woldetsadik, Miliyard Demeke Tafese, Biniyam Tefera Deressa, Chaojie Liu

**Affiliations:** ^1^ School of Psychology and Public Health La Trobe University Bundoora Victoria Australia; ^2^ Department of Human Anatomy, School of Medicine, College of Medicine and Health Sciences University of Gondar Gondar Ethiopia; ^3^ School of Population and Global Health University of Melbourne Melbourne Victoria Australia; ^4^ Department of Oncology, College of Health Sciences Addis Ababa University Addis Ababa Ethiopia; ^5^ Jimma Oncology Center Jimma University Jimma Ethiopia; ^6^ Adama Hospital Medical College Adama Ethiopia

**Keywords:** breast cancer, delay in cancer care, developing countries, Ethiopia

## Abstract

**Background:**

Breast cancer remains a major public health concern in Ethiopia, with timely access to care critical for improving outcomes. This study examined factors associated with delays in the breast cancer care pathway.

**Methods:**

This cross‐sectional study collected data from 458 women with histologically confirmed breast cancer between July and September 2024 at three tertiary hospitals. Data were collected through structured interviews and clinical records. Five time intervals from symptom detection to treatment initiation were analyzed using the Model of Pathways to Treatment. Factors were categorized using Andersen's Behavioral Model, and Accelerated Failure Time (AFT) models were used to estimate time ratios (TRs) with 95% confidence intervals (CIs).

**Results:**

Rural residence (TR = 1.84; 95% CI: 1.20–2.80) and painless breast mass (TR = 1.94; 95% CI: 1.26–3.00) were linked to longer delays from symptom recognition to first healthcare contact. Low breast cancer literacy and consulting traditional healers before diagnosis were consistently associated with prolonged delays. Consulting ≥ 3 providers extended both intervals, from first healthcare contact to diagnosis (TR = 3.13; 95% CI: 1.96–4.98) and symptom detection to diagnosis (TR = 1.63; 95% CI: 1.02–2.62). Delays were also associated with first providers failing to suspect or refer for cancer. Longer diagnosis‐to‐treatment intervals were observed among unmarried women, low‐income groups, and those using traditional healing post‐diagnosis. Older women (≥ 60 years) experienced shorter delays in initiating treatment (TR = 0.62; 95% CI: 0.41–0.93).

**Conclusion:**

Delays in breast cancer care in Ethiopia are driven by individual, sociocultural, and systemic barriers. Multilevel interventions are needed to promote early detection and timely treatment.

**Abbreviations**
FHCP: first health care provider
HCP: health care provider
HFCSH: Hiwot Fana Comprehensive Specialized Hospital
JUMC: Jimma University Medical Center
LMICs: low‐ and middle‐income countries
NCCP: National Cancer Control Plan

## 1 Introduction

Breast cancer remains a major public health challenge globally, especially in low‐ and middle‐income countries (LMICs), with disproportionately high mortality rates ranging from 40% to 60% [1, 2]. The primary reasons for higher mortality were thought to be limited access to cancer care and delayed diagnosis and treatment [2].

In Ethiopia, breast cancer accounts for a significant burden of cancer morbidity and mortality among women [3]. Breast cancer is a potentially treatable cancer if diagnosed at an early stage [4, 5]. Timely detection and initiation of treatment are critical for improving survival outcomes and minimizing the long‐term complications associated with advanced cancer therapies and expenses [6, 7]. Nevertheless, delays at various stages of the care continuum, from symptom recognition to diagnosis and treatment initiation continue to pose significant global healthcare challenges, often resulting in disease progression to advanced stages, reduced treatment effectiveness, and a shift in treatment intent from curative to palliative [8, 9].

The World Health Organization emphasizes the importance of empowering communities and enhancing health literacy as a critical first step toward promoting early cancer diagnosis [10]. However, in many LMICs, cancer literacy remains limited. Evidence from several studies indicates that poor health literacy is associated with prolonged diagnostic and treatment intervals [11, 12]. In LMICs, the resources and infrastructure for routine screening mammography are often unavailable [13]. The uptake of preventive screening measures remains limited in LMICs due to low cancer literacy and resource constraints [14].

In LMICs like Ethiopia, breast cancer patients frequently experience prolonged delays, contributing to a higher burden of advanced‐stage disease and mortality [2, 15, 16]. Research has identified a range of individual and contextual factors influencing delays at different stages [2, 17, 18]. Using Andersen's Behavioral Model, these delays can be understood through predisposing factors (such as low symptom awareness, sociocultural beliefs) [18, 19, 20, 21], enabling factors (including limited geographic access, financial hardship, lack of health insurance, and weak referral systems) [18, 22, 23], and need‐related factors (such as painless breast lump, presence of palpable axillary node, poor health literacy, and coexisting illnesses) [24]. Despite the recognized importance of early diagnosis and treatment, the LMICs face profound disparities in cancer care access due to socioeconomic, geographic, and educational barriers [14]. Socioeconomic inequality plays a pivotal role: Women from poorer and rural backgrounds often encounter greater obstacles to timely care due to structural disadvantages, limited health literacy, and constrained financial and logistical resources [25, 26].

In response to the growing burden of breast cancer, Ethiopia developed its first National Cancer Control Plan (NCCP) (2016–2020). The NCCP aimed to strengthen cancer prevention, improve early detection, particularly for cervical and breast cancers, and expand access to treatment and palliative care services [27]. Besides, recently, in 2024 the Ministry of Health launched the National Breast Cancer Control Guideline, promoting early detection through breast self‐awareness and clinical breast examination [28]. These national initiatives reflect a growing policy focus on reducing diagnostic and treatment delays; however, delays in care for women with breast cancer in Ethiopia continue to be a major public health concern.

At present, timely detection and treatment offer the best chance of improving breast cancer outcomes, and, consequently, are a key focus of breast cancer management and care [29]. Improving timely diagnosis and treatment first requires identifying the factors that contribute to unnecessary delay. A better understanding of factors associated with each stage of delay, is essential to design targeted interventions for earlier detection, faster diagnosis, and prompt initiation of care that can improve survival in Ethiopian women. However, most existing studies in Ethiopia focus on a single narrowed interval; for instance, the interval between symptom detection and first HCP contact, or are limited to specific settings, leaving critical knowledge gaps across the full cancer care pathway. Therefore, this study aimed to assess the factors influencing the entire patient care journey from symptom detection to treatment initiation among breast cancer patients in Ethiopia. The findings of this study will provide critical evidence to inform national cancer control strategies by identifying key factors contributing to delays in breast cancer care. The results can guide policymakers in designing targeted interventions to promote timely diagnosis and treatment, strengthen referral pathways, and improve access to early detection services, ultimately contributing to better breast cancer outcomes in Ethiopia.

## 2 Methods

### 2.1 Study Design and Population

This study employed a quantitative cross‐sectional design. Data were collected between July and September 2024 as part of a larger cross‐sectional study conducted among women diagnosed with breast cancer at three tertiary hospitals in Ethiopia. A total of 458 women diagnosed with breast cancer were consecutively recruited from patients attending or admitted to the oncology units of the participating hospitals. The study included women aged 18 years and older who had a histologically confirmed diagnosis of breast cancer, had initiated any form of treatment, and provided written informed consent to participate. Exclusion criteria were a prior history of mental illness, critical illness at the time of data collection, diagnosis with another concurrent malignancy, or a relapse of previously treated breast cancer.

### 2.2 Study Setting

Three major public hospitals in Ethiopia that provide comprehensive breast cancer services, including radiotherapy, were identified: Black Lion Hospital (Addis Ababa), Jimma University Medical Center (Jimma) (JUMC), and Hiwot Fana Comprehensive Specialized Hospital (Harar) (HFCSH). Black Lion Hospital, Ethiopia's national oncology hub, has more than 800 inpatient beds and serves an estimated 370,000–500,000 patients annually. Its oncology department accommodates approximately 60,000 outpatient visits and is staffed by eight oncologists with a 32‐bed inpatient oncology ward. JUMC, the primary cancer center in southwestern Ethiopia, has 800 beds and serves over 15 million people. The cancer center provides care to about 2500 patients annually, including 1000 receiving radiotherapy, and is staffed by five oncologists with 25 dedicated oncology beds. HFCSH, the main referral hospital in eastern Ethiopia, serves an estimated 16 million people. Its Ali Birra Memorial Cancer Center manages roughly 2000 cancer cases each year and is supported by three oncologists and 19 inpatient oncology beds.

The Ethiopian health system is structured in a three‐tier model: primary care units (health posts and health centers), secondary hospitals, and tertiary hospitals. The primary health care unit (PHCU) comprises a health post, health center and primary hospital. A single PHCU serves a total of 100,000 population. A general hospital serving about 500,000 population represents the secondary health care level while the specialized referral hospitals with a catchment population of 1000,000 are labeled tertiary health care level. While breast cancer care is formally included in Ethiopia's noncommunicable disease strategy, in practice, access to cancer diagnosis and treatment is highly centralized, with very few tertiary centers offering oncology services. Radiotherapy is limited to these three hospitals, which are functioning as the national oncology center and consistently providing radiotherapy and chemotherapy treatment services currently available at 24 public hospitals nationwide.

Human resources for cancer care are scarce, with a limited number of oncologists, pathologists, and trained oncology nurses nationwide. Currently, for a population of over 114 million people, there are close to 50 clinical oncologists trained to provide specialized cancer care to patients in Ethiopia [28]. Most health facilities at the lower levels of care lack the capacity to diagnose or manage cancer [30]. Ethiopia's health financing system is predominantly out‐of‐pocket (OOP) at the point of service. While Community‐Based Health Insurance has been introduced, cancer care is often inadequately covered, forcing patients and families to bear the cost of diagnostic imaging, pathology, chemotherapy drugs, and transport to treatment centers [31].

### 2.3 Data Collection Tools and Procedures

Data for this study were collected using an interviewer‐administered structured questionnaire and a review of patient medical records. The data collection process and tools were developed and implemented as part of a broader breast cancer research project previously described in a separate submitted manuscript. In brief, the questionnaire was developed in Redcap based on prior literature, national cancer care guidelines, and local contextual understanding. Three female medical doctors, trained for this purpose and not involved in the clinical care of participants, conducted face‐to‐face interviews during routine patient visits.

The tool captured detailed information on sociodemographic characteristics, clinical characteristics, date of symptom recognition, diagnosis and treatment, help‐seeking behavior, and timepoints relevant to the breast cancer care continuum [32]. Specific techniques were employed to reduce recall bias, including anchoring patient‐reported dates to local holidays and other calendrically memorable events.

To ensure completeness, date‐specific information on symptom onset, healthcare contact, diagnosis, and treatment initiation was triangulated from both patient response and clinical files. Regular supervision was conducted by the principal investigator, and a random 5% of entries were cross‐validated to ensure data quality.

### 2.4 Variables and Measurements

#### 2.4.1 Outcome and Exposure Variables

The primary outcomes of this study were the durations of care intervals from symptom detection to treatment initiation, measured in days. These intervals were operationalized based on the Model of Pathways to Treatment. The patient interval (PI) was defined as the number of days from a patient's first recognition of breast cancer symptoms to their initial contact with a healthcare provider. The diagnostic interval (DI) captured the time from first healthcare contact to the date of histological diagnosis, while the treatment interval (TI) represented the duration from confirmed diagnosis to the initiation of definitive cancer treatment (surgery, chemotherapy, or radiotherapy). Additionally, two cumulative measures were included: the total diagnostic interval (TDI), defined as the duration from symptom detection to diagnosis, and the total treatment interval (TTI), measuring the full time range from symptom onset to treatment initiation [33].

These time points were defined operationally as follows: “symptom detection” referred to the date the woman first noticed an abnormal breast change or lump; “first healthcare provider contact” was the date of the initial consultation with any qualified health professional; “diagnosis” referred to the date of histopathological (biopsy) confirmation; and “treatment initiation” was the date the first definitive cancer treatment (surgery, chemotherapy, or radiotherapy) commenced.

To identify determinants of delay, different covariates were considered, and we applied Andersen's Behavioral Model of Health Service Utilization as a conceptual framework [34]. This model categorizes explanatory variables into predisposing, enabling, and need‐related factors (Table 1). Among those variables, traditional healing indicates that the participants used holy water, visited traditional healers, or performed any ritual activities to treat breast cancer before and/or after diagnosis. Chronic illness was defined as a condition that lasts 1 year or more and requires ongoing medical attention or limits activities of daily living, or both; for example, heart disease, hypertension, cancer, and diabetes [35]. Medically diagnosed chronic illness was categorized into absent (0) or present (≥ 1) of any chronic illness other than cancer.

**TABLE 1:** Categorization of exposure variables by care interval based on Andersen's Behavioral Model of Health Services use.

Interval	Predisposing factors	Enabling factors	Need factors	Environmental/contextual enabling factors
PI: From symptom detection to first contact of HCP	Age, educational attainment, marital status, occupational status	Monthly household income, health insurance, traditional healing (before diagnosis), family size	Medically diagnosed chronic illness, family history of breast cancer, painless breast mass as first symptom, breast cancer literacy	Study site, distance to facility, residence
DI: From first contact of HCP to diagnosis	Age, educational attainment, marital status, occupational status	Monthly household income, health insurance, traditional healing (before diagnosis), family size, first contact with health care providers (HCP), additional HCP consultations	Medically diagnosed chronic illness, family history of breast cancer, painless breast mass as first symptom, breast cancer literacy	Study site, distance to facility, residence, first HCP suspected cancer, first HCP referral
TI: From diagnosis to treatment initiation	Age, educational attainment, marital status, occupational status	Monthly household income, health insurance, traditional healing (before and after diagnosis), family size, first contact with health care providers (HCP), additional HCP consultations	Medically diagnosed chronic illness, family history of breast cancer, painless breast mass as first symptom, breast cancer literacy, cancer stage at diagnosis	Study site, distance to facility, residence, first HCP suspected cancer, first HCP referral
TDI: From symptom detection to diagnosis	Age, educational attainment, marital status, occupational status	All enabling factors from PI and DI	All need‐related factors from PI and DI	All contextual enabling factors from PI and DI
TTI: From symptom detection to treatment initiation	Age, educational attainment, marital status, occupational status	All enabling factors from PI, DI, and TI	All need‐related factors from PI, DI, and TI	All contextual enabling factors from PI, DI, and TI

Abbreviations: DI, diagnostic interval; HCP, health care provider; PI, patient interval; TDI, total diagnostic interval; TI, treatment interval; TTI, total treatment interval.

Number of additional HCP consultations with formal care providers before (or after) diagnosis: number of healthcare professionals a patient visited before (or after) being formally diagnosed with breast cancer.

A family history of breast cancer was defined as having a first‐degree or second‐degree relative who had breast cancer.

Monthly household income was self‐reported by participants and based on the distribution of responses, income was categorized into tertiles:

- Low (Tertile 1): ≤ 2100 Ethiopian Birr (ETB)
- Medium (Tertile 2): 2101–3254 ETB
- High (Tertile 3): 3255–15,000 ETB

A composite breast cancer literacy score was used in the multivariable analysis to capture participants' awareness and understanding of the presentations of breast cancer, self‐examination, and screening measures, which involved five items. The score was constructed using five binary items: (1) whether the participant had ever heard about breast cancer, (2) knowledge of breast self‐examination (BSE), (3) regular performance of BSE, (4) awareness or prior use of mammography, and (5) correct interpretation of the first symptom. Participants were asked to choose “yes” (coded as 1), “don't know” or “no” (coded as 0) for each item. A summed score was calculated, ranging from 0 to 5, with a higher score indicating higher breast cancer literacy.

### 2.5 Data Analysis

All data analyses were conducted using Stata version 18. Descriptive statistics were used to summarize participant characteristics, with categorical variables presented as frequencies and percentages. Continuous variables were presented as median and interquartile range (IQR) based on the distribution of the data. To examine factors associated with the length of time across each defined interval from symptom detection to treatment initiation, we initially employed Cox proportional hazards models. However, due to the nonfulfillment of the proportional hazards assumption in some intervals, we subsequently applied Accelerated Failure Time (AFT) models. Given the positively skewed distribution of the time‐to‐event data, AFT models were used to accommodate the skewed nature of the data. Three AFT models were considered based on different distributional assumptions: Weibull, lognormal, and log‐logistic [36]. The best‐fitting distribution for each interval was selected based on the lowest Akaike Information Criterion and Bayesian Information Criterion values, and the highest log‐likelihood [37], detail presented in the Table S1. Consequently, the Weibull model was used for the interval from symptom detection to first HCP contact (PI) and log‐logistic for the rest of the intervals. To accommodate zero values in the time‐to‐event outcome variables and ensure compatibility with the log‐transformation required by AFT models, a small constant (0.5 days) was added to all time intervals with zero duration. Results from the AFT models are presented as Time Ratios (TRs) with 95% Confidence Intervals (CIs). A TR > 1 indicates a longer interval compared to the reference group, while a TR < 1 indicates a shorter interval. Both bivariate and multivariable analyses were conducted. All exposure variables were included in the multivariable model and in the analysis, a *p* value of < 0.05 was considered statistically significant. Multicollinearity was assessed using the Variance Inflation Factor (VIF). All variables had VIF values below 5, indicating no significant multicollinearity and acceptable independence among predictors in the model [38].

## 3 Results

### 3.1 Time Intervals in Breast Cancer Care by Sociodemographic Characteristics of Study Participants

**FIGURE 1:**
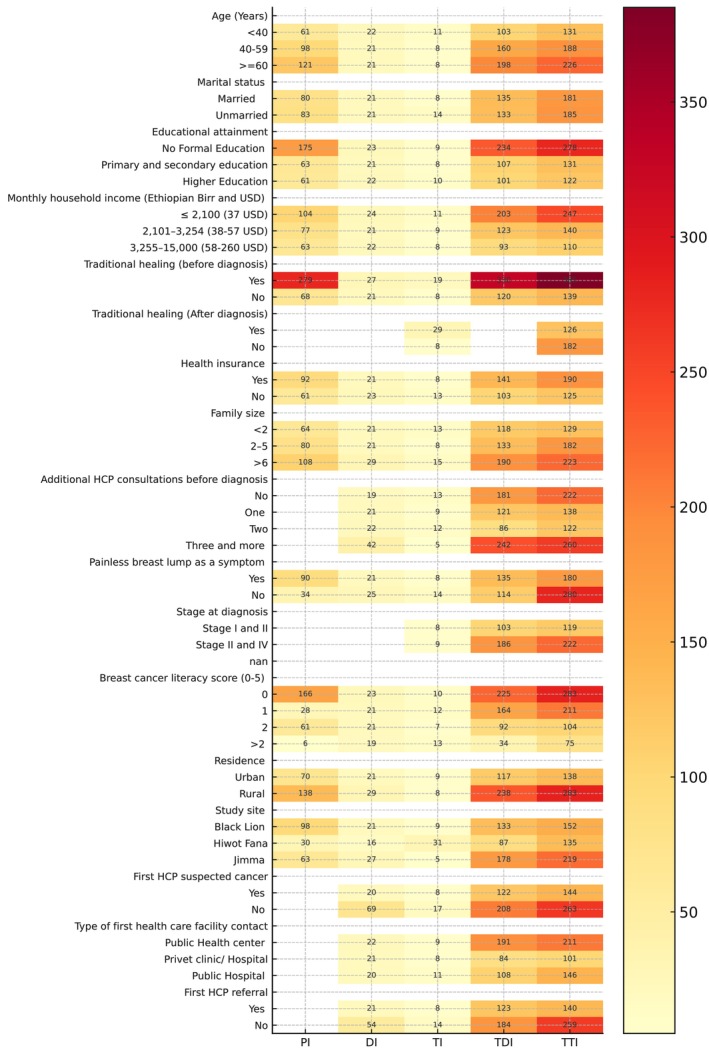
Comprehensive heatmap of median breast cancer care intervals (PI, DI, TI, TDI, and TTI).

This study included 458 women diagnosed with breast cancer. The majority were aged 40–59 years (51.8%), married (62.0%), and had low breast cancer literacy (63.1%); the score of each item is provided in the Table S2. The study found wide variations in breast cancer care intervals based on patient characteristics. Older women, those with low breast cancer literacy, lower income, rural residence, and those who used traditional healing reported longer median time intervals. For example, the median time from symptom detection to treatment (TTI) was 283 days for the poorest group compared to 121 days for the wealthiest. Women with a zero score in breast cancer literacy had a median TTI of 283 days versus 75 days for those with a score higher than 2. Use of traditional healing before diagnosis presented with a much longer median TTI (385 days vs. 139 days). Rural women also faced longer delays (median TTI: 283 days) compared to urban women (median: 138 days) (Figure 1). Additionally, patients whose first healthcare provider did not suspect cancer or did not refer them experienced longer diagnostic and treatment intervals. Overall, the median values consistently showed that socioeconomic and contextual disadvantages were linked with prolonged intervals in breast cancer care (Table 2).

**TABLE 2:** Characteristics of study participants and time intervals in seeking breast cancer care.

Category (Andersen's Model)	Variables	*n* (%)	Median PI (IQR)	Median DI (IQR)	Median TI (IQR)	Median TDI (IQR)	Median TTI (IQR)
Predisposing Factor	**Age (Years)**						
	< 40	168 (36.7)	61 (13–186)	22 (15–51)	11 (5–40)	103 (53–293)	131 (78–385)
	40–59	237 (51.8)	98 (17–222)	21 (15–35)	8 (5–22)	160 (66–329)	188 (84–374)
	≥ 60	53 (11.5)	121 (14–344)	21 (17–35)	8 (4–21)	198 (84–405)	226 (92–454)
	**Marital status**						
	Married	284 (62.0)	80 (14–217)	21 (15–42)	8 (5–21)	135 (57–33)	181 (77–379)
	Unmarried	174 (38.0)	83 (20–231)	21 (15–40)	14 (6–35)	133 (63–339)	185 (89–386)
	**Educational attainment**						
	No formal education	152 (33.1)	175 (58–339)	23 (16–46)	9 (5–26)	234 (113–392)	278 (126–431)
	Primary and secondary education	208 (45.4)	63 (8–188)	21 (12.5–35)	8 (5–28)	107 (46–300)	131 (69–363)
	Higher education	98 (21.4)	61 (13–121)	22 (15–44)	10 (5–25)	101 (52–210)	122 (67–257)
Enabling factor	**Monthly household income (Ethiopian Birr and USD)**						
	≤ 2100 (37 USD)	183 (40.0)	104 (8–287)	24 (8–71)	11 (4–35)	203 (87–369)	247 (117–401)
	2101–3254 (38–57 USD)	161 (35.1)	77 (19–231)	21 (17–30)	9 (6–25)	123 (59–351)	140 (75–388)
	3255–15,000 (58–260 USD)	114 (24.9)	63 (14–148)	22 (17–26)	8 (6–17)	93 (48–202)	110 (65–223)
	**Traditional healing (before diagnosis)**						
	Yes	59 (12.9)	279 (92–403)	27 (15–49)	19 (7–57)	330 (139–450)	385 (208–478)
	No	399 (87.1)	68 (13–188)	21 (15–40)	8 (5–24)	120 (54–291)	139 (75–342)
	**Traditional healing (After diagnosis)**						
	Yes	27 (5.90)	—	—	29 (9–184)	—	126 (42–342)
	No	421 (94.1)	—	—	8 (5–24)	—	182 (84–385)
	**Health insurance**						
	Yes	367 (80.1)	92 (18–239)	21 (15–40)	8 (5–25)	141 (63–350)	190 (85–387)
	No	91 (19.9)	61 (10–173)	23 (14–44)	13 (6–35)	103 (35–265)	125 (64–342)
	**Family size**						

	< 2	96 (21.0)	64 (16–198)	21 (16–36)	13 (6–31)	118 (58–294)	129 (79–342)
	2–5	267 (64.8)	80 (14–239)	21 (15–36)	8 (5–21)	133 (58–339)	182 (78–386)
> 6	65 (14.2)	108 (10–213)	29 (8–87)	15 (4–44)	190 (100–361)	223 (135–387)
	**Additional HCP consultations before diagnosis**						
	No	89 (19.4)	—	19 (15–27)	13 (6–44)	181 (75–339)	222 (104–401)
	One	162 (35.4)	—	21 (19–27)	9 (7–21)	121 (69–308)	138 (82–356)
	Two	100 (21.8)	—	22 (8–47)	12 (4–31)	86 (31–232)	122 (55–282)
	Three and more	107 (23.4)	—	42 (9–165)	5 (5–21)	242 (100–409)	260 (116–455)
Need factors	**Painless breast lump as a symptom**						
	Yes	409 (89.3)	90 (8–182)	21 (15–38)	8 (5–25)	135 (61–335)	180 (84–385)
	No	49 (10.7)	34 (15–234)	25 (10–98)	14 (6–52)	114 (43–339)	280 (59–381)
	**Stage at diagnosis**						
	Stage I and II	189 (41.2)	—	—	8 (6–24)	103 (48–238)	119 (62–307)
	Stage II and IV	269 (58.8)	—	—	9 (5–29)	186 (82–373)	222 (105–339)
	**Breast cancer literacy score (0–5)**						
	0	143 (31.2)	166 (49–344)	23 (15–44)	10 (5–33)	225 (96–392)	283 (128–429)
	1	146 (31.9)	28 (35–239)	21 (15–49)	12 (6–33)	164 (75–350)	211 (107–399)
	2	112 (24.5)	61 (14–109)	21 (18–27)	7 (5–14)	92 (53–199)	104 (66–221)
	> 2	57 (12.6)	6 (1–61)	19 (8–56)	13 (4–28)	34 (14–181)	75 (21–223)
Contextual factor	**Residence**						
	Urban	339 (74.0)	70 (14–195)	21 (15–30)	9 (6–26)	117 (54–300)	138 (70–352)
	Rural	119 (26.0)	138 (18, 333)	29 (8–87)	8 (3–31)	238 (96–391)	283 (126–424)
	**Study site**						
	Black Lion	252 (55.0)	98 (42–294)	21 (19–28)	9 (7–21)	133 (73–335)	152 (85–377)
	Hiwot Fana	65 (14.2)	30 (6–134)	16 (6–55)	31 (7–83)	87 (21–218)	135 (51–343)
	Jimma	141 (30.8)	63 (3–218)	27 (7–121)	5 (3–21)	178 (59–380)	219 (86–394)

	**First HCP suspected cancer**						
	Yes	360 (78.6)	——	20 (14–27)	8 (7–44)	122 (57–325)	144 (77–371)
	No	98 (21.4)	——	69 (33–199)	17 (5–22)	208 (87–378)	263 (118, 404)
	**Type of first health care facility contact**						
	Public Health center	250 (54.6)	—	22 (18–36)	9 (6–28)	191 (89–365)	211 (110–401)
	Privet clinic/Hospital	88 (19.2)	—	21 (14–39)	8 (5–21)	84 (40–206)	101 (57–255)
	Public Hospital	120 (26.2)	—	20 (6–55)	11 (4–30)	108 (31–342)	146 (62–387)
	**First HCP referral**						
	Yes	326 (71.1)	—	21 (15–27)	8 (5–21)	123 (55–320)	140 (75–354)
No	132 (28.9)	—	54 (14–155)	14 (5–44)	184 (83–378)	259 (108–417)

*Note:* 1US$ = 57.7.

The multivariable AFT regression results for all five care intervals are presented in Table 3.

**TABLE 3:** Summary table of multivariable analysis of factors associated with time intervals in breast cancer care in Ethiopia (*N* = 458).

Variables	PI TR (95% CI)	DI TR (95% CI)	TI TR (95% CI)	TDI TR (95% CI)	TTI TR (95% CI)	VIF
Predisposing factors						
Age (Years)						
< 40	Ref	Ref	Ref	Ref	Ref	
40–59	0.96 (0.71–1.29)	0.99 (0.81–1.22)	0.87 (0.68, 1.12)	1.08 (0.85, 1.36)	1.00 (0.82, 1.23)	1.40
≥ 60	1.08 (0.64–1.81)	0.97 (0.69–1.35)	0.62 (0.41, 0.93)*	1.17 (0.80, 1.72)	1.06 (0.75, 1.50)	1.63
Educational attainment						
No formal education	1.61 (0.99–2.60)	1.04 (0.75–1.44)	1.00 (0.67, 1.49)	1.39 (0.96, 2.03)	1.20 (0.86, 1.68)	3.50
Primary & Secondary	1.18 (0.80–1.73)	0.84 (0.65–1.08)	0.97 (0.71, 1.31)	0.97 (0.72, 1.31)	0.95 (0.73, 1.23)	2.34
Higher education	Ref	Ref	Ref	Ref	Ref	
Marital status						
Married	Ref	Ref	Ref		Ref	
Unmarried	1.08 (0.81–1.44)	0.99 (0.81–1.21)	1.46 (1.14, 1.87)**	1.08 (0.85, 1.36)	1.14 (0.92, 1.39)	1.35
Occupational status						
Employed	Ref	Ref	Ref	Ref	Ref	
Unemployed	0.93 (0.67–1.29)	1.11 (0.89–1.38)	1.18 (0.91, 1.53)	0.88 (0.68, 1.14)	0.92 (0.68, 1.14)	168
Enabling factors						
Monthly household income (Ethiopian Birr)						
≤ 2100 (37 USD)	1.06 (0.68–1.64)	0.78 (0.59–1.04)	1.48 (1.05, 2.08)*	1.11 (0.80, 1.54)	1.15 (0.86, 1.54)	2.69
2101–3254 (38–57 USD)	1.05 (0.73–1.50)	0.99 (0.79–1.24)	1.29 (0.98, 1.70)	1.12 (0.85, 1.48)	1.14 (0.90, 1.45)	1.82
3255–15,000 (58–260 USD)	Ref	Ref	Ref	Ref	Ref	
Health insurance						
Yes	Ref	Ref	Ref	Ref	Ref	
No	0.80 (0.58–1.12)	0.98 (0.78–1.22)	1.02 (0.77, 1.35)	0.83 (0.64, 1.08)	0.84 (0.67, 1.08)	1.16
Use of traditional healing before diagnosis						
Yes	2.21 (1.50–3.26)**,*	1.13 (0.85–1.48)	1.34 (0.96, 1.87)	2.14 (1.60, 2.87)***	1.84 (1.42, 2.39)***	1.17
No	Ref					
Use of traditional healing after diagnosis						
Yes	—	—	2.25 (1.33, 3.80)**	—	0.98 (0.66, 1.47)	1.16
No	—	—	Ref	—	Ref	
Family size						
< 2	Ref	Ref	Ref	Ref		
2–5	0.96 (0.68–1.34)	0.95 (0.75–1.20)	0.98 (0.74, 1.30)	1.00 (0.76, 1.32)	1.05 (0.83, 1.33)	1.79
≥ 6	0.98 (0.60–1.60)	0.99 (0.68–1.46)	1.34 (0.86, 2.07)	1.12 (0.74, 1.69)	1.05 (0.72, 1.52)	2.12
First health care facility contacts						
Public hospital	—	Ref	Ref	Ref	Ref	
Public health center	—	1.07 (0.83–1.38)	0.86 (0.64, 1.16)	1.29 (0.98, 1.72)	1.15 (0.90, 1.47)	1.94
Privet hospital/clinic	—	1.00 (0.75–1.34)	0.96 (0.68, 1.35)	0.96 (0.69, 1.34)	0.96 (0.67, 1.20)	1.69
Additional HCP consultations						
None	—	Ref	Ref	Ref	Ref	
One	—	1.02 (0.80–1.30)	0.89 (0.65, 1.20)	0.78 (0.59, 1.04)	0.78 (0.60, 1.01)	2.11
Two	—	1.18 (0.88–1.59)	0.83 (0.58, 1.19)	0.81 (0.57, 1.13)	0.75 (0.55, 1.01)	2.11
Three or more		3.13 (1.96–4.98)***	0.92 (0.54, 1.56)	1.63 (1.02, 2.62)*	1.33 (0.88, 2.01)	4.01
Need factors						
Family history of breast cancer						
Yes	Ref					
No	1.65 (0.93–2.93)	0.88 (0.60–1.30)	1.41 (0.89, 2.25)	1.02 (0.66, 1.58)	1.21 (0.82, 1.80)	1.15
Medically diagnosed chronic illness						
Yes	0.90 (0.63–1.28)	1.05 (0.84–1.32)	1.14 (0.86, 1.50)	0.99 (0.77, 1.28)	1.04 (0.83, 1.31)	1.18
No	Ref	Ref	Ref	Ref	Ref	
Painless breast mass as first symptom						
Yes	1.95 (1.26–3.00)**	0.86 (0.62–1.20)	0.83 (0.57, 1.21)	1.14 (0.81, 1.60)	1.06 (0.79, 1.45)	1.14
No	Ref	Ref	Ref	Ref		
Stage of cancer						
Stage I & II	—	—	Ref	—		
Stage III or IV	—	—	1.02 (0.81, 1.28)	—	1.34 (1.11, 1.63)**	1.25
Breast cancer literacy score (0–5)						
0	2.83 (1.67–4.79)***	1.14 (0.78–1.67)	1.06 (0.68, 1.64)	2.47 (1.60, 3.81)***	1.98 (1.35, 2.89)***	3.74
1	2.69 (1.67–4.34)***	1.08 (0.77–1.52)	1.15 (0.78, 1.69)	2.38 (1.61, 3.51)***	1.91 (1.38, 2.45)***	3.01
2	1.90 (1.18–3.06)**	1.13 (0.80–1.58)	0.80 (0.54, 1.18)	1.87 (1.26, 2.78)**	1.43 (1.01, 2.06)*	2.62
> 2	Ref	Ref	Ref	Ref	Ref	
Contextual enabling factors						
Residence						
Rural	1.84 (1.20, 2.80)**	1.09 (0.80–1.50)	0.90 (0.62, 1.30)	1.18 (0.84, 1.66)	1.07 (0.79, 1.46)	2.27
Urban	Ref	Ref	Ref	Ref	Ref	
Study site						
Black Lion Hospital	2.26 (1.43–3.57)***	2.09 (1.47–2.99)***	0.63 (0.41, 0.98)*	2.58 (1.75, 3.79)***	2.58 (1.74, 3.79)**	3.79
Jimma Hospital	1.09 (0.66–1.81)	0.94 (0.60–1.48)	0.26 (0.16, 0.44)***	1.51 (0.96, 2.38)	1.51 (0.73, 2.38)	4.21
Hiwot Fana Hospital	Ref	Ref	Ref	Ref	Ref	
Distance to the nearest health facility (km)						
< 5 km	Ref	Ref	Ref	Ref	Ref	
≥ 5 km	1.23 (0.87–1.75)	0.93 (0.75–1.17)	0.82 (0.63, 1.06)	0.95 (0.74, 1.21)	0.91 (0.74, 1.21)	1.27
First HCP referral						
Yes	—	Ref	Ref	Ref	Ref	
No	—	1.36 (0.99–1.88)	1.15 (0.81, 1.63)	1.47 (1.06, 2.03)*	1.47 (1.11, 1.95)*	2.12
HCP suspected cancer						
Yes	—	Ref	Ref	Ref	Ref	
No	—	3.07 (2.15–4.39)***	1.32 (0.90, 1.94)	1.06 (0.75, 1.50)	1.05 (0.77, 1.42)	2.11

*Note:* *p < 0.05; **p < 0.01; ***p < 0.00.

Abbreviations: 95% CI, 95% confidence interval; TR, time ratio; VIF, Variance Inflation Factor.

### 3.2 Factors Associated With Patient Interval (PI) Between Symptom Recognition and First Contact With a Healthcare Provider

Women residing in rural areas experienced a significantly longer PI compared to their urban counterparts (TR = 1.84; 95% CI: 1.20–2.80; *p* = 0.005). Participants from Black Lion Hospital also had substantially longer PI (TR = 2.26; 95% CI: 1.43–3.57; *p* < 0.001) compared to those from Hiwot Fana. Seeking traditional healing prior to diagnosis was associated with a twofold increase in the duration of PI (TR = 2.21; 95% CI: 1.50–3.26; *p* < 0.001). Breast cancer literacy was a strong predictor of PI: Lower cancer literacy scores were associated with longer PI, with TRs ranging from 1.90 to 2.83 (*p* < 0.01). Finally, reporting a painless breast lump as the initial symptom was associated with a nearly twofold increase in PI (TR = 1.95; 95% CI: 1.26–3.00; *p* = 0.003).

### 3.3 Factors Associated With DI From First Contact With Health Care Providers to Diagnosis

Women who consulted three or more healthcare providers prior to diagnosis experienced a substantially longer DI (TR = 3.13; 95% CI: 1.96–4.98; *p* < 0.001). Similarly, patients whose symptoms were initially not suspected to be cancer by the first healthcare provider had a markedly longer DI (TR = 3.07; 95% CI: 2.15–4.39; *p* < 0.001). Additionally, patients seen at Black Lion Hospital experienced significantly longer DI (TR = 2.09; 95% CI: 1.47–2.99; *p* < 0.001) compared to those presenting at Hiwot Fana Hospital.

### 3.4 Factors Associated With Treatment Interval (TI) From Diagnosis to Treatment Initiation

Women aged ≥ 60 years experienced significantly shorter TI compared to those under 40 (TR = 0.62; 95% CI: 0.41–0.93; *p* = 0.021). Conversely, TI was significantly longer among unmarried women (TR = 1.46; 95% CI: 1.14–1.87; *p* = 0.002) and those from the lowest‐income (≤ 1200 BT) households (TR = 1.48; 95% CI: 1.05–2.08; *p* = 0.026), compared to their respective counterparts. Notably, patients presenting at Jimma University Hospital (TR = 0.26; 95% CI: 0.16–0.44; *p* < 0.00) and Black Lion Hospital (TR = 0.63; 95% CI: 0.41–0.98; *p* = 0.038) had significantly shorter TI compared to those at Hiwot Fana Hospital. Importantly, women who sought help from traditional healers after receiving a diagnosis had a 2.25‐fold longer TI (TR = 2.25; 95% CI: 1.33–3.80; *p* = 0.003) than those who did not.

### 3.5 Factors Associated With TDI Between Symptom Recognition and Diagnosis

Breast cancer literacy was a significant predictor of the interval between symptom detection and diagnosis. Compared to women with high literacy scores, those with lower cancer literacy scores experienced a significantly longer TDI with TR ranging from 1.87 to 2.47 (*p* < 0.01). Women who had visited traditional healers before diagnosis experienced significantly prolonged TDI (TR = 2.14; 95% CI: 1.60–2.87; *p* < 0.001). Moreover, those who were presented at Black Lion Hospital, had longer TDI (TR = 2.58; 95% CI: 1.75–3.79; *p* < 0.001) compared to those at Hiwot Fana Hospital. Finally, consulting three or more healthcare providers before diagnosis was also associated with longer TDI (TR = 1.63; 95% CI: 1.02–2.62; *p* = 0.041) and patients who did not receive a referral from the first health care provider experienced prolonged TDI compared to those who were referred, with a TR of 1.47 (95% CI: 1.06–2.03; *p* = 0.019).

### 3.6 Factors Associated With Total Treatment Interval (TTI) From Symptom Recognition to Treatment Initiation

Breast cancer literacy was a significant predictor of TTI: Lower cancer literacy scores were associated with longer TTI, with TRs ranging from 1.43 to 1.98 (*p* < 0.001). Women who visited traditional healers prior to diagnosis were also strongly associated with TTI (TR = 1.84; 95% CI: 1.42–2.39; *p* < 0.001). Compared to patients diagnosed at Hiwot Fana Hospital, those diagnosed at Black Lion Hospital had longer TTI (TR = 2.58; 95% CI: 1.74–3.79; *p* = 0.011). Patients who were not referred by their first healthcare provider had prolonged TTI compared to those who were referred (TR = 1.47; 95% CI: 1.11–1.95; *p* = 0.008). Additionally, women diagnosed at stages III and IV had longer TTI (TR = 1.34; 95% CI: 1.11–1.63; *p* = 0.003).

Full details of the bivariate and multivariable analyses for all five outcome variables are presented in Table S3.

## 4 Discussion

This study comprehensively examined factors influencing the timeliness of breast cancer care in Ethiopia by analyzing five key time intervals across the care continuum, interpreted through Andersen's Behavioral Model of Health Services Utilization. Several sociodemographic, clinical, and system‐level factors were identified as predictors of longer intervals. Policymakers, healthcare professionals and other stakeholders can use the insights from this study to develop practical strategies to mitigate delays in presentation, diagnosis, and treatment of breast cancer.

### 4.1 Predisposing Factors

Predisposing factors, including age and marital status, emerged as important determinants. Unmarried women experienced prolonged time to initiate cancer treatment following diagnosis. This aligns with a study in Sub‐Saharan Africa which indicates that social and spousal support play a crucial role in navigating complex cancer care pathways; unmarried women often experience weaker social networks, fewer caregivers, and more limited emotional and logistical support, which can hinder timely access to care [39].

### 4.2 Enabling Factors

Enabling factors, such as household income, consultation with multiple healthcare providers prior to diagnosis, and the use of traditional healing, significantly influenced time intervals. Women from low‐income households experienced longer intervals between diagnosis and treatment initiation, largely due to financial constraints that limit their ability to afford both the direct and indirect costs of cancer care. This is consistent with systematic reviews and empirical studies from LMICs, which have highlighted poverty and financial hardship as key drivers of delayed diagnosis and treatment [2, 40, 41]. In resource‐limited settings like Ethiopia, families often prioritize basic survival needs over timely medical care, reinforcing income‐related inequities in access and timeliness [2, 16, 41]. Interestingly, in our study, health insurance coverage did not significantly reduce delays, despite approximately 80% of women reporting having health insurance. This contrasts with evidence suggesting that community‐based or national health insurance can improve service utilization and financial protection in Ethiopia [31]. The lack of association in our sample may reflect limited benefit packages for oncology services, gaps in awareness of entitlements, administrative barriers, or high out‐of‐pocket expenses that persist despite nominal coverage [30]. These findings indicate that insurance expansion alone is insufficient; the depth and scope of coverage, particularly for cancer diagnostics and treatment, must be strengthened to achieve meaningful improvements in timeliness.

The use of traditional healing before diagnosis was strongly associated with longer intervals from symptom detection to initial healthcare contact, diagnosis, and treatment initiation. Post‐diagnosis use of traditional healing was also linked to extended intervals between diagnosis and treatment initiation. In Ethiopia, where traditional healing is used by approximately 65% of the population [42], patients often turn to nonmedical care due to financial hardship [43, 44], limited accessibility of modern health services [43], or beliefs that cancer is incurable through conventional medicine [30, 45]. Studies in Ethiopia and other African countries have similarly reported that these practices delay engagement with the formal health system and contribute to advanced‐stage presentation [46, 47, 48, 49].

Furthermore, women who consulted three or more healthcare providers prior to diagnosis experienced substantially longer intervals from their first healthcare contact to diagnosis, as well as from symptom detection to diagnosis. This highlights the impact of fragmented referral processes within Ethiopia's health system [50]. Similar patterns have been documented in African and Asian settings, where patients circulate between primary care facilities, private clinics, and traditional healers before receiving a definitive diagnosis [18, 49, 51].

### 4.3 Need‐Related Factors

Need‐related factors, such as breast cancer literacy, symptom type, and stage at diagnosis are also associated with delays. Women with lower breast cancer literacy experienced significantly longer intervals from symptom detection to both diagnosis and treatment initiation. This finding is consistent with systematic reviews and primary studies across LMICs, which show that poor knowledge of breast cancer symptoms, risk factors, and the benefit of early detection is a major barrier to timely presentation [52, 53, 54]. Knowledge of breast cancer, screening and early detection methods, and initial symptoms, is essential for recognizing early signs and seeking timely medical care [51, 55]. However, in this study, 63% of women scored zero on the breast cancer literacy measure, suggesting that many fail to recognize symptoms or underestimate their severity. Consequently, care is often sought only when symptoms worsen or when physical discomfort begins to significantly disrupt their daily lives [24]. Additionally, limited understanding of the importance of timely care may lead to missed appointments, poor provider communication, and further delays.

A painless breast lump, the most common initial symptom, was associated with longer care‐seeking delays, likely because patients underestimated its seriousness. This finding is consistent with previous studies conducted in Ethiopia [56], Egypt [57], and a systematic review of evidence from sub‐Saharan African countries [58], all of which highlight that the absence of pain often contributes to delayed presentation among breast cancer patients. Our findings confirm that symptom appraisal is a critical phase in the Model of Pathways to Treatment [33]. Women diagnosed at advanced stages also experienced longer delays from symptom detection to treatment initiation. The finding is consistent with studies from LMICs showing that late‐stage disease is both a consequence and a marker of prolonged patient and system delays [23, 46, 50].

### 4.4 Contextual Factors

Contextual factors, including place of residence, study site, first HCP referral, and cancer suspicion played a critical role. Rural residence was linked to a longer patient interval, reflecting access disparities in Ethiopia. This finding is consistent with previous studies conducted in South Africa [59], Morocco [60], and Ethiopia [24, 56], which consistently show that women from rural areas face transport barriers, long travel distances, lower income, and poorer access to health information, all of which contribute to delayed presentation. In Ethiopia, patients from rural areas often have low awareness of the importance of timely health‐seeking behavior and face significant barriers to accessing healthcare services and information. These women frequently encounter transportation challenges and must travel long distances to reach health facilities. Furthermore, limited health insurance coverage and low household income in rural settings contribute to the unaffordability of cancer care, ultimately leading to delays in presentation.

Variation across the three study sites in Ethiopia, also influenced care timeliness, likely due to differences in capacity and service availability. Patients at Black Lion Hospital experienced longer delays in presentation and diagnosis, likely due to high patient volumes and its role as a national referral center. It is also likely that many patients seek care at Black Lion Hospital as a last resort, after delays at lower‐level facilities, contributing to longer timelines observed in the care pathway. As the national oncology hub, Black Lion Hospital receives complex cases and referrals from across the country, which can lead to overcrowding, long waiting times for appointments and pathology, and delays in diagnostic and treatment pathways. Interestingly, despite these delays, patients at Black Lion Hospital had shorter intervals between diagnosis and treatment initiation. Accessing services at Black Lion Hospital is challenging for many patients; however, once admitted, the hospital is able to deliver care efficiently. This may be explained by recent institutional improvements, including the establishment of a “one‐stop” breast cancer care center that facilitates immediate post‐diagnosis treatment planning and initiation. Establishing similar integrated centers in other regional hospitals could strengthen timely breast cancer care in Ethiopia. Additionally, the hospital's private wing, where oncologists offer services outside of regular hours, may expedite access to surgery and other treatments. Notably, over 80% of patients in this study started treatment with surgery, and the presence of many surgical residents likely supports more timely surgical scheduling compared to other sites.

At Jimma University Medical Center, patients faced longer DIs, but shorter treatment intervals compared to Hiwot Fana Hospital. This pattern may reflect variations in diagnostic infrastructure and oncologist availability. While resource constraints may delay pathology results at Jimma, its higher number of oncologists than Hiwot Fana Hospital could support faster treatment initiation post‐diagnosis. These findings highlight how site‐level differences in capacity and workflow can shape care timeliness in resource‐limited settings. Comparable site‐level variations in timeliness have been documented in Brazil, South Africa, and other LMIC settings, where differences in workforce, equipment, and institutional processes shape patients' trajectories [41, 46].

Patients not referred by their first HCP experienced a longer interval from symptom detection to diagnosis and treatment initiation, highlighting the importance of a proper referral pathway. This is attributed to the lack of expertise in the health facility or proper guidelines for referrals. Besides, the first HCP's failure to suspect cancer was associated with prolonged diagnosis. Previous literature has reported that misdiagnosis, underestimation of symptoms, and inappropriate initial management at primary‐level facilities are key contributors to delay [45, 61, 62]. Misdiagnosis is frequently linked to inadequate knowledge and insufficient training of health care workers regarding breast cancer. In our study more than half of the patients (54.6%) visited health centers; these centers are often staffed by health officers, nurses, or health assistants who may not have adequate training in breast cancer recognition and treatment guidelines. This can lead to failure to suspect cancer at the initial consultation (misdiagnosis), underestimation of symptoms, or inappropriate referrals, ultimately resulting in delayed diagnostic confirmation [63, 64]. To address this, health worker capacity in early detection must be strengthened, and standardized referral mechanisms should be developed. Diagnostic checklists could also help ensure symptoms are systematically investigated.

Gender norms in Ethiopia may also influence delays, as male partners often hold decision‐making authority. Limited female autonomy and stigma around breast symptoms can discourage timely care‐seeking. Although not measured in our study, these sociocultural dynamics likely contribute to delayed presentation.

The findings underscore that delays in breast cancer care result from a complex interplay of individual, social, and health system factors. Addressing these delays requires coordinated governance and policy‐level action. Strengthening referral systems through clear national guidelines and improving coordination between primary, secondary, and tertiary care levels is essential to reduce delays. Expanding diagnostic capacity at lower‐level facilities, training health workers in clinical breast examination, and integrating breast cancer awareness into primary health care can facilitate earlier detection. Community education to improve breast cancer literacy, alongside the strategic engagement of traditional healers in referral pathways, could further enhance early presentation. To address financial and geographic barriers, especially for low‐income populations, it is critical to expand health insurance coverage, provide subsidized treatment options, and implement accessible, equity‐focused policies. Scaling up integrated “one‐stop” cancer clinics and developing regional diagnostic and treatment centers are practical, system‐level actions that align with Ethiopia's National Cancer Control Plan and the WHO Global Breast Cancer Initiative goals for timely diagnosis and care.

This study's strengths include its comprehensive analysis of five time intervals in the care pathway and use of Andersen's Behavioral Model to interpret multilevel influences. The study was conducted across three major oncology centers, enhancing generalizability within Ethiopia. However, as a cross‐sectional study, causal relationships cannot be established. The reliance on patient self‐report for timing of symptom onset and early healthcare contact introduces the possibility of recall bias. Additionally, because the sample includes only patients who accessed tertiary care facilities, the experiences of women who were unable to reach such centers, potentially due to the most severe access barriers, are not captured, which may limit the findings' generalizability to all breast cancer patients. We were unable to include the variable measuring distance from patients' homes to cancer centers, which may have influenced care‐seeking behavior and delays. Another limitation is the inability to determine the exact facility where each patient received their diagnosis. Despite these limitations, the study offers important insights into the drivers of delay in breast cancer care and provides actionable recommendations to improve timely presentation, diagnosis, and treatment in Ethiopia.

## 5 Conclusion

This study demonstrates that delays in breast cancer care in Ethiopia are driven by a complex interplay of predisposing, enabling, need‐related, and contextual factors. Women with low breast cancer literacy and those who were unmarried experienced longer delays, underscoring the importance of awareness and social support in timely care‐seeking. Financial constraints, reliance on traditional healing and visiting multiple healthcare providers before receiving a diagnosis were significant contributors, reflecting the impact of economic, cultural barriers and system‐level barriers. Clinically, the presence of a painless breast lump and advanced‐stage disease at presentation were associated with delayed care, highlighting how symptom perception influences health‐seeking behavior. Contextual factors such as rural residence, the study site, whether the first healthcare provider made a referral, and whether cancer was initially suspected by the first provider all influenced the timeliness of care. Delays were more common among those who were not referred by the initial provider, or where cancer was not initially suspected. Together, these findings emphasize the need for multifaceted interventions that strengthen early detection, improve referral pathways, enhance provider training, and address financial and geographic barriers to ensure timely and equitable breast cancer care in Ethiopia.

## Author Contributions

**Anteneh Ayelign Kibret:** conceptualization, methodology, formal analysis, software, data curation, investigation, writing – original draft, writing – review and editing. **Jason Jiang:** conceptualization, writing – review and editing, supervision, methodology. **Edom Seife Woldetsadik:** supervision, writing – review and editing. **Miliyard Demeke Tafese:** writing – review and editing, supervision. **Biniyam Tefera Deressa:** supervision, writing – review and editing. **Chaojie Liu:** conceptualization, methodology, supervision, writing – review and editing.

## Funding

The authors have nothing to report.

## Ethics Statement

Participation in the study was entirely voluntary. Institutional permission was obtained to access medical records. All potential participants received detailed information about the study's objectives and procedures and provided written informed consent prior to the interview. Ethical approval was obtained from the Human Ethics Committee of La Trobe University, Melbourne, Australia (HEC 24025), and the Clinical Oncology Department of Black Lion Hospital, Addis Ababa, Ethiopia (Ethics/003/2016).

## Conflicts of Interest

The authors declare no conflicts of interest.

## Supporting information


**Table S1:** Model Fit Statistics for Time Intervals in Breast Cancer Care.

**Table S2:** Breast cancer literacy assessment.

**Table S3:** cam471439‐sup‐0003‐TableS3.docx.

## Data Availability

The datasets generated and/or analyzed during this study are not publicly available due to the inclusion of potentially identifiable health information. Data may be made available upon reasonable request, subject to approval by the La Trobe University Human Research Ethics Committee. Interested researchers should contact Professor George Liu (c.liu@latrobe.edu.au) or the Ethics Committee (humanethics@latrobe.edu.au).

## References

[1] H. Sung , J. Ferlay , R. L. Siegel , et al., “Global Cancer Statistics 2020: GLOBOCAN Estimates of Incidence and Mortality Worldwide for 36 Cancers in 185 Countries,” CA: A Cancer Journal for Clinicians 71, no. 3 (2021): 209–249. 33538338 10.3322/caac.21660

[2] M. M. Rivera‐Franco and E. Leon‐Rodriguez , “Delays in Breast Cancer Detection and Treatment in Developing Countries,” Breast Cancer: Basic and Clinical Research 12 (2018): 1178223417752677. 29434475 10.1177/1178223417752677 PMC5802601

[3] S. T. Memirie , M. K. Habtemariam , M. Asefa , et al., “Estimates of Cancer Incidence in Ethiopia in 2015 Using Population‐Based Registry Data,” Journal of Global Oncology 4 (2018): 1–11. 10.1200/JGO.17.00175 PMC6223441 30241262

[4] O. Ginsburg , C. H. Yip , A. Brooks , et al., “Breast Cancer Early Detection: A Phased Approach to Implementation,” Cancer 126, no. Suppl 10 (2020): 2379–2393. 32348566 10.1002/cncr.32887 PMC7237065

[5] L. Wang , “Early Diagnosis of Breast Cancer,” Sensors (Basel) 17, no. 7 (2017): 1572. 28678153 10.3390/s17071572 PMC5539491

[6] T. P. Hanna , W. D. King , S. Thibodeau , et al., “Mortality due to Cancer Treatment Delay: Systematic Review and Meta‐Analysis,” BMJ 371 (2020): m4087. 33148535 10.1136/bmj.m4087 PMC7610021

[7] J. M. McLaughlin , R. T. Anderson , A. K. Ferketich , E. E. Seiber , R. Balkrishnan , and E. D. Paskett , “Effect on Survival of Longer Intervals Between Confirmed Diagnosis and Treatment Initiation Among Low‐Income Women With Breast Cancer,” Journal of Clinical Oncology 30, no. 36 (2012): 4493–4500. 23169521 10.1200/JCO.2012.39.7695 PMC3518728

[8] J. Zhang , M. IJzerman , and J. D. Emery , “Timely Cancer Diagnosis and Treatment: Towards a Generalisable Research Framework Studying Timeliness to Appropriate Care,” Annals of Cancer Epidemiology 7 (2023): 3.

[9] Q. Huo , C. Cai , Y. Zhang , et al., “Delay in Diagnosis and Treatment of Symptomatic Breast Cancer in China,” Annals of Surgical Oncology 22 (2015): 883–888. 25212834 10.1245/s10434-014-4076-9

[10] Organization WH , WHO Report on Cancer: Setting Priorities, Investing Wisely and Providing Care for All (World Health Organization, 2020).

[11] C. E. Jones , J. Maben , R. H. Jack , et al., “A Systematic Review of Barriers to Early Presentation and Diagnosis With Breast Cancer Among Black Women,” BMJ Open 4, no. 2 (2014): e004076. 10.1136/bmjopen-2013-004076 PMC3927711 24523424

[12] L. Forbes , F. Warburton , M. Richards , and A. Ramirez , “Risk Factors for Delay in Symptomatic Presentation: A Survey of Cancer Patients,” British Journal of Cancer 111, no. 3 (2014): 581–588. 24918824 10.1038/bjc.2014.304 PMC4119978

[13] A. Tfayli , S. Temraz , R. Abou Mrad , and A. Shamseddine , “Breast Cancer in Low‐ and Middle‐Income Countries: An Emerging and Challenging Epidemic,” Journal of Oncology 2010, no. 1 (2010): 490631. 21209708 10.1155/2010/490631 PMC3010663

[14] O. A. Bamodu and C.‐C. Chung , “Cancer Care Disparities: Overcoming Barriers to Cancer Control in Low‐ and Middle‐Income Countries,” JCO Global Oncology 10 (2024): e2300439. 39173080 10.1200/GO.23.00439

[15] Y. M. Martei , L. E. Pace , J. E. Brock , and L. N. Shulman , “Breast Cancer in Low‐ and Middle‐Income Countries: Why we Need Pathology Capability to Solve This Challenge,” Clinics in Laboratory Medicine 38, no. 1 (2018): 161–173. 29412880 10.1016/j.cll.2017.10.013 PMC6277976

[16] O. Ginsburg , A. Rositch , L. Conteh , M. Mutebi , E. Paskett , and S. Subramanian , “Breast Cancer Disparities Among Women in Low‐and Middle‐Income Countries,” Current Breast Cancer Reports 10 (2018): 179–186.

[17] L. A. Hoveling , M. Schuurman , S. Siesling , K. M. van Asselt , and C. Bode , “Diagnostic Delay in Women With Cancer: What Do We Know and Which Factors Contribute?,” Breast 80 (2025): 104427. 39987718 10.1016/j.breast.2025.104427 PMC11904510

[18] A. Afaya , S. Ramazanu , O. A. Bolarinwa , et al., “Health System Barriers Influencing Timely Breast Cancer Diagnosis and Treatment Among Women in Low and Middle‐Income Asian Countries: Evidence From a Mixed‐Methods Systematic Review,” BMC Health Services Research 22, no. 1 (2022): 1601. 36587198 10.1186/s12913-022-08927-x PMC9805268

[19] A. G. Q. Freitas and M. Weller , “Patient Delays and System Delays in Breast Cancer Treatment in Developed and Developing Countries,” Ciência & Saúde Coletiva 20 (2015): 3177–3189. 26465859 10.1590/1413-812320152010.19692014

[20] S. D. Malope , S. A. Norris , and M. Joffe , “Culture, Community, and Cancer: Understandings of Breast Cancer From a Non‐Lived Experience Among Women Living in Soweto,” BMC Women's Health 24, no. 1 (2024): 594. 39506786 10.1186/s12905-024-03431-2 PMC11539428

[21] T. De Ver Dye , S. Bogale , C. Hobden , et al., “A Mixed‐Method Assessment of Beliefs and Practice Around Breast Cancer in Ethiopia: Implications for Public Health Programming and Cancer Control,” Global Public Health 6, no. 7 (2011): 719–731. 20865612 10.1080/17441692.2010.510479

[22] S. Getachew , A. Tesfaw , M. Kaba , et al., “Perceived Barriers to Early Diagnosis of Breast Cancer in South and Southwestern Ethiopia: A Qualitative Study,” BMC Women's Health 20, no. 1 (2020): 38. 32103774 10.1186/s12905-020-00909-7 PMC7045514

[23] E. Jedy‐Agba , V. McCormack , O. Olaomi , et al., “Determinants of Stage at Diagnosis of Breast Cancer in Nigerian Women: Sociodemographic, Breast Cancer Awareness, Health Care Access and Clinical Factors,” Cancer Causes & Control 28 (2017): 685–697. 28447308 10.1007/s10552-017-0894-y PMC5492222

[24] D. E. Wendimu , M. B. Degefa , D. L. Achalu , B. T. Mamo , D. B. Daba , and S. G. Meshesha , “Timeliness of Breast Cancer Patients' Presentation to Health Care Facilities in Ethiopia: A Systematic Review and Meta‐Analysis,” JCO Global Oncology 10 (2024): e2400263. 39571108 10.1200/GO-24-00263

[25] R. U. Osarogiagbon , H. M. Sineshaw , J. M. Unger , A. Acuña‐Villaorduña , and S. Goel , “Immune‐Based Cancer Treatment: Addressing Disparities in Access and Outcomes,” American Society of Clinical Oncology Educational Book American Society of Clinical Oncology Annual Meeting 41 (2021): 1–13. 10.1200/EDBK_323523 33830825

[26] M. I. Patel , A. M. Lopez , W. Blackstock , et al., “Cancer Disparities and Health Equity: A Policy Statement From the American Society of Clinical Oncology,” Journal of Clinical Oncology 38, no. 29 (2020): 3439–3448. 32783672 10.1200/JCO.20.00642 PMC7527158

[27] 2016‐2020 FMOHE , “National Cancer Control Plan 2015,” https://www.iccp‐portal.org/system/files/plans/NCCP%20Ethiopia%20Final%20.pdf.

[28] Ethiopia MoH , “National Guideline for Breast Health, Early Diagnosis and Timely Breast Cancer Management In Ethiopia April, 2024,” https://www.iccp‐portal.org/system/files/resources/FINAL%20Breast%20Cancer%20Guideline%20%20Ethiopia%202024.pdf.

[29] S. S. Gorin , J. E. Heck , B. Cheng , and S. J. Smith , “Delays in Breast Cancer Diagnosis and Treatment by Racial/Ethnic Group,” Archives of Internal Medicine 166, no. 20 (2006): 2244–2252. 17101943 10.1001/archinte.166.20.2244

[30] W. Haileselassie , T. Mulugeta , W. Tigeneh , M. Kaba , and W. L. Labisso , “The Situation of Cancer Treatment in Ethiopia: Challenges and Opportunities,” J Cancer Prev 24, no. 1 (2019): 33–42. 30993093 10.15430/JCP.2019.24.1.33 PMC6453587

[31] F. D. Bayou , M. Arefaynie , Y. Tsega , et al., “Effect of Community Based Health Insurance on Healthcare Services Utilization in Ethiopia: A Systematic Review and Meta‐Analysis,” BMC Health Services Research 24, no. 1 (2024): 1188. 39369193 10.1186/s12913-024-11617-5 PMC11456236

[32] A. A. Kibret , H. Jiang , H. Yang , and C. Liu , “Patient Journey and Timeliness of Care for Patients With Breast Cancer in Africa: A Scoping Review Protocol,” BMJ Open 14, no. 9 (2024): e081256. 10.1136/bmjopen-2023-081256 PMC11381707 39242165

[33] S. Scott , F. Walter , A. Webster , S. Sutton , and J. Emery , “The Model of Pathways to Treatment: Conceptualization and Integration With Existing Theory,” British Journal of Health Psychology 18, no. 1 (2013): 45–65. 22536840 10.1111/j.2044-8287.2012.02077.x

[34] A. Alkhawaldeh , M. ALBashtawy , A. Rayan , et al., “Application and Use of Andersen's Behavioral Model as Theoretical Framework: A Systematic Literature Review From 2012–2021,” Iranian Journal of Public Health 52, no. 7 (2023): 1346–1354. 37593505 10.18502/ijph.v52i7.13236 PMC10430393

[35] CDC , “About Chronic Diseases October 4, 2024,” https://www.cdc.gov/chronic‐disease/about/index.html?utm_source=chatgpt.com.

[36] E. T. Lee and J. Wang , Statistical Methods for Survival Data Analysis (John Wiley & Sons, 2003).

[37] A. Othman and S. Hasan , “Application of the Accelerated Failure Time Model to Lung Cancer Data,” International Journal of Nonlinear Analysis and Applications (IJNAA) 12 (2021): 1243–1250.

[38] R. M. O'brien , “A Caution Regarding Rules of Thumb for Variance Inflation Factors,” Quality and Quantity 41, no. 5 (2007): 673–690.

[39] R. Yuan , C. Zhang , Q. Li , M. Ji , and N. He , “The Impact of Marital Status on Stage at Diagnosis and Survival of Female Patients With Breast and Gynecologic Cancers: A Meta‐Analysis,” Gynecologic Oncology 162, no. 3 (2021): 778–787. 34140180 10.1016/j.ygyno.2021.06.008

[40] R. Subedi , N. Houssami , C. Nickson , et al., “Factors Influencing the Time to Diagnosis and Treatment of Breast Cancer Among Women in Low‐and Middle‐Income Countries: A Systematic Review,” Breast 75 (2024): 103714. 38522173 10.1016/j.breast.2024.103714 PMC10973645

[41] N. Alves Soares Ferreira , M. Figueiredo , S. de Carvalho , et al., “Treatment Delays Among Women With Breast Cancer in a Low Socio‐Economic Status Region in Brazil,” BMC Women's Health 17, no. 1 (2017): 13. 28222726 10.1186/s12905-016-0359-6 PMC5320774

[42] N. Tuasha , S. Fekadu , and S. Deyno , “Prevalence of Herbal and Traditional Medicine in Ethiopia: A Systematic Review and Meta‐Analysis of 20‐Year Studies,” Systematic Reviews 12, no. 1 (2023): 232. 38093343 10.1186/s13643-023-02398-9 PMC10717384

[43] S. Kumar Pal , “Use of Alternative Cancer Medicine in India,” Lancet Oncology 3, no. 7 (2002): 394–395. 12142166 10.1016/s1470-2045(02)00782-9

[44] P. Tovey , J. Chatwin , and S. Ahmad , “Toward an Understanding of Decision Making on Complementary and Alternative Medicine Use in Poorer Countries: The Case of Cancer Care in Pakistan,” Integrative Cancer Therapies 4, no. 3 (2005): 236–241. 16113031 10.1177/1534735405278641

[45] A. Gebremariam , A. Addissie , A. Worku , M. Assefa , E. J. Kantelhardt , and A. Jemal , “Perspectives of Patients, Family Members, and Health Care Providers on Late Diagnosis of Breast Cancer in Ethiopia: A Qualitative Study,” PLoS One 14, no. 8 (2019): e0220769. 31369640 10.1371/journal.pone.0220769 PMC6675093

[46] J. Moodley , L. Cairncross , T. Naiker , and D. Constant , “From Symptom Discovery to Treatment‐Women's Pathways to Breast Cancer Care: A Cross‐Sectional Study,” BMC Cancer 18 (2018): 1–11. 29562894 10.1186/s12885-018-4219-7 PMC5863383

[47] M. Tessema Ersumo , M. Girmaye Tamrat , M. Bogale Solomon , and M. Tariku Gero , “Breast Cancer in a Private Medical Services Center: A 10‐Year Experience,” Ethiopian Medical Journal 35 (2018): 35–44.

[48] T. D. Dye , S. Bogale , C. Hobden , Y. Tilahun , T. Deressa , and A. Reeler , “Experience of Initial Symptoms of Breast Cancer and Triggers for Action in Ethiopia,” International Journal of Breast Cancer 2012, no. 1 (2012): 908547. 22315692 10.1155/2012/908547 PMC3270501

[49] O. Agodirin , I. Aremu , G. Rahman , et al., “Determinants of Delayed Presentation and Advanced‐Stage Diagnosis of Breast Cancer in Africa: A Systematic Review and Meta‐Analysis,” Asian Pacific Journal of Cancer Prevention 22, no. 4 (2021): 1007–1017. 33906291 10.31557/APJCP.2021.22.4.1007 PMC8325140

[50] A. Gebremariam , A. Addissie , A. Worku , et al., “Time Intervals Experienced Between First Symptom Recognition and Pathologic Diagnosis of Breast Cancer in Addis Ababa, Ethiopia: A Cross‐Sectional Study,” BMJ Open 9, no. 11 (2019): e032228. 10.1136/bmjopen-2019-032228 PMC6858206 31719089

[51] C. Webber , L. Jiang , E. Grunfeld , and P. A. Groome , “Identifying Predictors of Delayed Diagnoses in Symptomatic Breast Cancer: A Scoping Review,” European Journal of Cancer Care 26, no. 2 (2017): e12483. 10.1111/ecc.12483 26950652

[52] D. Petrova , D. Garrido , Z. Špacírová , et al., “Duration of the Patient Interval in Breast Cancer and Factors Associated With Longer Delays in Low‐and Middle‐Income Countries: A Systematic Review With Meta‐Analysis,” Psycho‐Oncology 32, no. 1 (2023): 13–24. 36345154 10.1002/pon.6064 PMC10100001

[53] O. Agodirin , I. Aremu , G. Rahman , et al., “Determinants of Delayed Presentation and Advanced‐Stage Diagnosis of Breast Cancer in Africa: A Systematic Review and Meta‐Analysis,” Asian Pacific Journal of Cancer Prevention: APJCP 22, no. 4 (2021): 1007–1017. 33906291 10.31557/APJCP.2021.22.4.1007 PMC8325140

[54] S. Alhurishi , J. Lim , B. Potrata , and R. West , “Factors Influencing Late Presentation for Breast Cancer in the Middle East: A Systematic Review,” Asian Pacific Journal of Cancer Prevention 12, no. 6 (2011): 1597–1600. 22126505

[55] S. L. Bodapati and G. R. Babu , “Oncologist Perspectives on Breast Cancer Screening in India‐Results From a Qualitative Study in Andhra Pradesh,” Asian Pacific Journal of Cancer Prevention 14, no. 10 (2013): 5817–5823. 24289583 10.7314/apjcp.2013.14.10.5817

[56] A. Tesfaw , D. Solomon , M. Tigabu , and Z. Ashuro , “Patient Delay and Contributing Factors Among Breast Cancer Patients at Two Cancer Referral Centres in Ethiopia: A Cross‐Sectional Study,” Journal of Multidisciplinary Healthcare 13 (2020): 1391–1401. 33173301 10.2147/JMDH.S275157 PMC7646382

[57] S. M. Mousa , I. A. Seifeldin , A. Hablas , E. S. Elbana , and A. S. Soliman , “Patterns of Seeking Medical Care Among Egyptian Breast Cancer Patients: Relationship to Late‐Stage Presentation,” Breast 20, no. 6 (2011): 555–561. 21807518 10.1016/j.breast.2011.07.001 PMC4274941

[58] C. Espina , F. McKenzie , and I. dos‐Santos‐Silva , “Delayed Presentation and Diagnosis of Breast Cancer in African Women: A Systematic Review,” Annals of Epidemiology 27, no. 10 (2017): 659–671. 29128086 10.1016/j.annepidem.2017.09.007 PMC5697496

[59] S. Rayne , K. Schnippel , S. Grover , D. Kruger , C. Benn , and C. Firnhaber , “The Effect of Beliefs About Breast Cancer on Stage and Delay to Presentation: Results From a Prospective Study in Urban South Africa,” South African Journal of Surgery 57, no. 1 (2019): 12–18.

[60] B. Benbakhta , M. Tazi , N. Benjaafar , A. Khattabi , and A. Maaroufi , “Determinants of Patient and Health System Delays for Women With Breast Cancer in Morocco, 2013,” Revue D'epidemiologie et de Sante Publique 63, no. 3 (2015): 191–201. 10.1016/j.respe.2015.03.121 25975777

[61] H. Rastad , N. Khanjani , and B. K. Khandani , “Causes of Delay in Seeking Treatment in Patients With Breast Cancer in Iran: A Qualitative Content Analysis Study,” Asian Pacific Journal of Cancer Prevention 13, no. 9 (2012): 4511–4515. 23167370 10.7314/apjcp.2012.13.9.4511

[62] A. Poum , S. Promthet , S. W. Duffy , and D. M. Parkin , “Factors Associated With Delayed Diagnosis of Breast Cancer in Northeast Thailand,” Journal of Epidemiology 24, no. 2 (2014): 102–108. 24335087 10.2188/jea.JE20130090 PMC3983282

[63] S. Getachew , A. Tesfaw , M. Kaba , et al., “Perceived Barriers to Early Diagnosis of Breast Cancer in South and Southwestern Ethiopia: A Qualitative Study,” BMC Women's Health 20 (2020): 1–8. 32103774 10.1186/s12905-020-00909-7 PMC7045514

[64] R. E. Kohler , S. Gopal , A. R. Miller , et al., “A Framework for Improving Early Detection of Breast Cancer in Sub‐Saharan Africa: A Qualitative Study of Help‐Seeking Behaviors Among Malawian Women,” Patient Education and Counseling 100, no. 1 (2017): 167–173. 27528411 10.1016/j.pec.2016.08.012 PMC5301948

