# Supplementary material for: Factors Influencing Time to Treatment Initiation for Breast Cancer in Ethiopia
Source: Cancer Med. 2025 Dec 4;14(23):e71439. doi: 10.1002/cam4.71439 (PMC12678031; doi:10.1002/cam4.71439)
Supplement: Supplementary file 1 — Table S1: Model Fit Statistics for Time Intervals in Breast Cancer Care. [file CAM4-14-e71439-s001.docx]

# Supplementary Table 1. Model Fit Statistics for Time Intervals in Breast Cancer Care

This tables presents the log-likelihood, Akaike Information Criterion (AIC), and Bayesian Information Criterion (BIC) for three parametric accelerated failure time (AFT) models (Weibull, Lognormal, and Loglogistic) fitted separately for each of the five time intervals in the breast cancer care continuum:
- Patient Interval (PI)
- Diagnostic Interval (DI)
- Treatment Interval (TI)
- Total Diagnostic Interval (TDI)
- Time to Treatment Initiation (TTI)

Lower values of AIC and BIC indicate better model fit. The best-fitting model for each interval is highlighted in bold.

## Patient Interval (PI):

We compared three parametric AFT models, Weibull, lognormal, and loglogistic, using Akaike’s Information Criterion (AIC) and Bayesian Information Criterion (BIC). The Weibull model demonstrated the best overall fit (AIC = 1764.23; BIC = 1863.28) compared to the lognormal (AIC = 1766.70) and loglogistic (AIC = 1775.29) models and was therefore selected for final analysis.

Table 1: Model Fit Statistics for Parametric AFT Models of Time to First Healthcare Contact

| **Model** | **Log-Likelihood** | **AIC** | **BIC** |
| --- | --- | --- | --- |
| **Weibull** | **-858.115** | **1764.23** | **1863.28** |
| Lognormal | -859.350 | 1766.70 | 1865.75 |
| Loglogistic | -863.646 | 1775.29 | 1874.34 |

## Diagnostic Interval (DI):

The loglogistic model provides the best fit for modeling the time from pathological confirmation to treatment, based on both AIC and BIC. Therefore, it is the most appropriate model for analyzing diagnostic interval in this study

Table 2: Model Fit Statistics for Parametric AFT Models of diagnostic interval

| **Model** | **Log-Likelihood** | **AIC** | **BIC** |
| --- | --- | --- | --- |
| **Loglogistic** | **-652.76** | **1367.53** | **1495.46** |
| Lognormal | -670.50 | 1403.01 | 1530.94 |
| Weibull | -703.27 | 1468.54 | 1596.48 |

## Treatment Interval (TI):

Among the three AFT models fitted for treatment interval, the **loglogistic model** provided the best fit, with the lowest Akaike Information Criterion (AIC = 1503.1) and Bayesian Information Criterion (BIC = 1639.3).

Table 3: Model Fit Statistics for Parametric AFT Models of treatment interval

| **Model** | **Log Likelihood** | **AIC** | **BIC** |
| --- | --- | --- | --- |
| **Loglogistic** | **-718.55** | **1503.09** | **1639.28** |
| Lognormal | -727.56 | 1521.12 | 1657.30 |
| Weibull | -781.39 | 1628.79 | 1764.98 |

## Total Diagnostic Interval (TDI):

Table 4: Model Fit Statistics for Parametric AFT Models of the interval between symptom detection and diagnosis

| Model | Log Likelihood | AIC | BIC |
| --- | --- | --- | --- |
| **Loglogistic** | **-687.803** | **1437.606** | **1565.539** |
| Lognormal | -691.616 | 1445.231 | 1573.164 |
| Weibull | -700.578 | 1463.156 | 1591.089 |

## Time to Treatment Initiation (TTI):

Table 5: Model Fit Statistics for Parametric AFT Models of the interval between symptom detection and treatment

| Model | Log Likelihood | AIC | BIC |
| --- | --- | --- | --- |
| Loglogistic | -626.904 | 1319.807 | 1455.994 |
| Lognormal | -631.263 | 1328.526 | 1464.713 |
| Weibull | -648.755 | 1363.51 | 1499.696 |

## Summary Interpretation

- The Weibull model provided the best fit for the Patient Interval

- For the Diagnostic, Treatment, Total Diagnostic, and Time to Treatment Initiation intervals, the Loglogistic model showed consistently superior performance, as indicated by the lowest AIC and BIC values.
- These findings informed the selection of the final AFT models used in multivariable analysis for each interval.
