# Supplementary material for: Factors Influencing Time to Treatment Initiation for Breast Cancer in Ethiopia
Source: Cancer Med. 2025 Dec 4;14(23):e71439. doi: 10.1002/cam4.71439 (PMC12678031; doi:10.1002/cam4.71439)
Supplement: Supplementary file 2 — Table S2: Breast cancer literacy assessment. [file CAM4-14-e71439-s002.docx]

# Supplementary file 2: Breast cancer literacy assessment

# Table 1: Distribution of breast cancer literacy responses by item (N = 458)

| Question | Yes (n, %) | No (n, %) |
| --- | --- | --- |
| Have you ever heard of breast cancer prior to your first symptom? | 268 (58.52%) | 190 (41.48%) |
| Did you suspect your symptom was cancer? | 46 (10.04%) | 412 (89.96%) |
| Do you know how to examine your breasts using your hand for lumps? | 200 (43.67%) | 258 (56.33%) |
| Before noticing symptoms, did you regularly examine your breasts? | 30 (6.55%) | 428 (93.45%) |
| Before noticing symptoms, did you have prior use of mammography? | 11 (2.40%) | 447 (97.60%) |

# Table 2: Breast cancer literacy score distribution

| Score | Frequency | Percent (%) | Cumulative Percent (%) |
| --- | --- | --- | --- |
| 0 | 143.0 | 31.22 | 31.22 |
| 1 | 146.0 | 31.88 | 63.1 |
| 2 | 112.0 | 24.45 | 87.55 |
| 3 | 46.0 | 10.04 | 97.6 |
| 4 | 8.0 | 1.75 | 99.34 |
| 5 | 3.0 | 0.66 | 100.0 |
