# Supplementary material for: Factors Influencing Time to Treatment Initiation for Breast Cancer in Ethiopia
Source: Cancer Med. 2025 Dec 4;14(23):e71439. doi: 10.1002/cam4.71439 (PMC12678031; doi:10.1002/cam4.71439)
Supplement: Supplementary file 3 — Table S3: cam471439‐sup‐0003‐TableS3.docx. [file CAM4-14-e71439-s003.docx]

**Supplementary file table 3: Depicts the full details of the bivariate and multivariable analyses for all five outcome variables**

Table 1: Bivariate and multivariable Weibull AFT Regression analysis of factors associated with longer time between symptom detection and first contact with health care providers among women with breast cancer in Ethiopia (N=458).

| Variable | Bivariate coefficient (95% CI) | P-value | Multivariable coefficient (95% CI) | TR (95% CI) | P-value |
| --- | --- | --- | --- | --- | --- |
| Age in years |  |  |  |  |  |
| <40 | Ref |  |  | Ref |  |
| 40-59 | 0.24 (-0.05, 0.53) | 0.112 | -0.04 (-0.34 – 0.25) | 0.96 (0.71 – 1.29) | 0.768 |
| >=60 | 0.616 (0.15, 1.07) | 0.009 | 0.07 (-0.45 – 0.59) | 1.08 (0.64 – 1.81) | 0.786 |
| Residence |  |  |  |  |  |
| Rural | **0.37(0.06, 0.68)** | **0.020** | **0.60 (0.18 – 1.03)** | **1.84 (1.20 – 2.80)** | **0.005** |
| Urban | Ref |  |  | Ref |  |
| Study site |  |  |  |  |  |
| Black Lion | **1.17 (0.68,1.67)** | **0.000** | **0.81 (0.35 – 1.27)** | **2.26 (1.43 – 3.57)** | **0.000** |
| Jimma | 0.02 (-0.51, 0.55) | 0.946 | 0.08 (-0.42 – 0.60) | 1.09 (0.66 – 1.81) | 0.741 |
| Hiwot Fana | Ref |  | Ref |  |  |
| Educational attainment |  |  |  |  |  |
| No formal education | 0.91 (0.54, 1.28) | 0.000 | 0.47 (-0.00 – 0.95) | 1.61 (0.99 – 2.60) | 0.054 |
| Primary & Secondary | 0.30 (-0.04, 0.65) | 0.089 | 0.16 (-0.22 – 0.55) | 1.18 (0.80 – 1.73) | 0.411 |
| Higher education | Ref |  |  | Ref |  |
| Marital status |  |  |  |  |  |
| Married | Ref |  |  | Ref |  |
| Unmarried | 0.09 (-0.19, 0.37) |  | 0.07(-0.21 – 0.36) | 1.08 (0.81 – 1.44) | 0.602 |
| Occupational Status |  |  |  |  |  |
| Employed | Ref |  |  | Ref |  |
| Unemployed | 0.39(0.12, 0.66) | 0.005 | -0.07 (-0.40– 0.25) | 0.93 (0.67 – 1.29) | 0.656 |
| Monthly household income per capita (ETB) |  |  |  |  |  |
| ≤2,100 (37 USD) | 0.30(0.001, 0.612) | 0.049 | 0.05 (-0.38 – 0.49) | 1.06 (0.68 – 1.64) | 0.800 |
| 2,101–3,254 (38-57 USD) | 0.34 (-0.03, 0.72) | 0.073 | 0.04 (-0.31 – 0.40) | 1.05 (0.73 – 1.50) | 0.810 |
| 3,255–15,000 (58-260 USD) | Ref |  |  | Ref |  |
| Health Insurance |  |  |  |  |  |
| Yes | Ref |  |  | Ref |  |
| No | -0.32 (-0.67, 0.02) | 0.065 | -0.21 (-0.55 – 0.11) | 0.80 (0.58 – 1.12) | 0.199 |
| Traditional medicine use before diagnosis |  |  |  |  |  |
| Yes | **0.95 (0.55, 1.35)** | **0.000** | **0.79 (0.40 – 1.18)** | **2.21 (1.50 – 3.26)** | **0.000** |
| No | Ref |  |  | Ref |  |
| Family History of Breast Cancer |  |  |  |  |  |
| Yes | Ref |  |  | Ref |  |
| No | 0.72 (0.14, 1.30) | 0.015 | 0.50 (-0.07 – 1.07) | 1.65 (0.93 – 2.93) | 0.087 |
| Distance to the nearest health facility(km) |  |  |  |  |  |
| <5km | Ref |  |  | Ref |  |
| >=5km | 0.17 (-0.15, 0.49) | 0.302 | 0.20 (-0.14 – 0.55) | 1.23 (0.87 – 1.75) | 0.246 |
| Breast cancer literacy (0-5) |  |  |  |  |  |
| 0 | **1.66 (1.21, 2.11)** | **0.000** | **1.04 (0.51 – 1.56)** | **2.83 (1.67 – 4.79)** | **0.000** |
| 1 | **1.30 (0.86, 1.75)** | **0.000** | **0.99 (0.51 – 1.46)** | **2.69 (1.67 – 4.34)** | **0.000** |
| 2 | **0.81 (0.35, 1.27)** | **0.000** | **0.64 (0.16 – 1.11)** | **1.90 (1.18 – 3.06)** | **0.009** |
| >2 | Ref |  | Ref |  |  |
| Medically Confirmed chronic illness |  |  |  |  |  |
| Yes | 0.18 (-0.17, 0.52) | 0.319 | -0.10(-0.45 – 0.24) | 0.90 (0.63 – 1.28) | 0.551 |
| No | Ref |  |  | Ref |  |
| Family size |  |  |  |  |  |
| <2 | Ref |  |  |  |  |
| 2-5 | 0.07 (-0.27, 0.42) | 0.668 | -0.04 (-0.38 – 0.29) | 0.96 (0.68 – 1.34) | 0.794 |
| ≥6 | -0.01 (-0.48, 0.45) | 0.952 | -0.02 (-0.51 – 0.46) | 0.98 (0.60 – 1.60) | 0.926 |
| Painless breast mass as first symptom |  |  |  |  |  |
| Yes | **0.59 (0.15, 1.03)** | **0.008** | **0.66 (0.23 – 1.10)** | **1.95 (1.26 – 3.00)** | **0.003** |
| No | Ref |  | Ref |  |  |

Table 2: Bivariate and multivariable Loglogistic AFT Regression analysis of factors associated with longer time between first contact with health care providers and diagnosis among Ethiopian breast cancer patients.

| Variable | Bivariate coefficient (95% CI) | P-value | Multivariable coefficient (95% CI) | TR (95% CI) | P-value |
| --- | --- | --- | --- | --- | --- |
| Age in years |  |  |  |  |  |
| <40 | Ref |  | Ref |  |  |
| 40-59 | -0.11 (-0.34, 0.10) | 0.302 | -0.01 (-0.21 – 0.20) | 0.99 (0.81 – 1.22) | 0.956 |
| >=60 | -0.09 (-0.43, 0.24) | 0.579 | -0.04 (-0.37 – 0.30) | 0.97 (0.69 – 1.35) | 0.834 |
| Residence |  |  |  |  |  |
| Rural | 0.30 (0.05, 0.56) | 0.017 | 0.09 (-0.23 – 0.40) | 1.09 (0.80 – 1.50) | 0.578 |
| Urban | Ref |  | Ref |  |  |
| Study site |  |  |  |  |  |
| Black Lion | **0.28 (-0.04, 0.61)** | **0.089** | **0.74 (0.38 – 1.10)** | **2.09 (1.47 – 2.99)** | **0.000** |
| Jimma | 0.40 (0.01, 0.78) | 0.041 | -0.06 (-0.52 – 0.40) | 0.94 (0.60 – 1.48) | 0.794 |
| Hiwot Fana | Ref |  | Ref |  |  |
| Educational attainment |  |  |  |  |  |
| No formal education | 0.03 (-0.24, 0.31) | 0.821 | 0.04 (-0.29 – 0.37) | 1.04 (0.75 – 1.44) | 0.815 |
| Primary & Secondary | -0.232 (-0.49, 0.03) | 0.086 | -0.18 (-0.43 – 0.07) | 0.84 (0.65 – 1.08) | 0.164 |
| Higher education | Ref |  | Ref |  |  |
| Marital status |  |  |  |  |  |
| Married | Ref |  | Ref |  |  |
| Unmarried | -0.02 (-0.23, 0.19) | 0.854 | -0.01 (-0.22 – 0.19) | 0.99 (0.81 – 1.21) | 0.910 |
| Occupational Status |  |  |  |  |  |
| Employed | Ref |  |  |  |  |
| Unemployed | 0.07 (-0.13, 0.28) | 0.466 | 0.10 (-0.12 – 0.32) | 1.11 (0.89 – 1.38) | 0.356 |
| Monthly income per capita (ETB) |  |  |  |  |  |
| ≤2,100 (37 USD) | 0.12 (-0.11, 0.35) | 0.308 | -0.25 (-0.53 – 0.04) | 0.78 (0.59 – 1.04) | 0.094 |
| 2,101–3,254 (38-57 USD) | 0.05 (-0.21, 0.32) | 0.692 | -0.01 (-0.24 – 0.22) | 0.99 (0.79 – 1.24) | 0.911 |
| 3,255–15,000 (58-260 USD) | Ref |  |  |  |  |
| Health Insurance |  |  |  |  |  |
| Yes | Ref |  | Ref |  |  |
| No | 0.10 (-0.15, 0.36) | 0.425 | -0.03 (-0.25 – 0.20) | 0.98 (0.78 – 1.22) | 0.831 |
| Distance to the nearest health facility(km) |  |  |  |  |  |
| <5 | Ref |  |  |  |  |
| >=5 | 0.06 (-0.18, 0.317) | 0.605 | -0.07 (-0.29 – 0.16) | 0.93 (0.75 – 1.17) | 0.553 |
| Traditional healing before diagnosis |  |  |  |  |  |
| Yes | 0.20 (-0.10, 0.50) | 0.191 | 0.12 (-0.16 – 0.40) | 1.13 (0.85 – 1.48) | 0.400 |
| No | Ref |  | Ref |  |  |
| Family History of breast cancer |  |  |  |  |  |
| Yes | Ref |  | Ref |  |  |
| No | -0.23 (-0.68, 0.21) | 0.300 | -0.13 (-0.51 – 0.26) | 0.88 (0.60 – 1.30) | 0.524 |
| Medically Confirmed chronic illness |  |  |  |  |  |
| Yes | -0.12 (-0.37, 0.12) | 0.313 | 0.05 (-0.18 – 0.28) | 1.05 (0.84 – 1.32) | 0.654 |
| No | Ref |  | Ref |  |  |
| Painless breast mass as first symptom |  |  |  |  |  |
| Yes | -0.17 (-0.55, 0.20) | 0.370 | -0.15 (-0.48 – 0.18) | 0.86 (0.62 – 1.20) | 0.383 |
| No | Ref |  | Ref |  |  |
| Additional HCP consultations |  |  |  |  |  |
| None | Ref |  | Ref |  |  |
| One | 0.17 (-0.08, 0.43) | 0.186 | 0.02 (-0.22 – 0.27) | 1.02 (0.80 – 1.30) | 0.854 |
| Two | 0.07 (-0.23, 0.38) | 0.637 | 0.17 (-0.13 – 0.46) | 1.18 (0.88 – 1.59) | 0.269 |
| Three and more | **0.81 (0.47, 1.14)** | **0.000** | **1.14 (0.68 – 1.61)** | **3.13 (1.96 – 4.98)** | **0.000** |
| First HCP referral |  |  |  |  |  |
| Yes | Ref |  | Ref |  |  |
| No | 0.89 (0.65, 1.13) | 0.000 | 0.31 (-0.01 – 0.63) | 1.36 (0.99 – 1.88) | 0.06 |
| Breast cancer literacy (0-5) |  |  |  |  |  |
| 0 | 0.32 (-0.04, 0.69) | 0.082 | 0.13 (-0.25 – 0.51) | 1.14 (0.78 – 1.67) | 0.500 |
| 1 | 0.26 (-0.10, 0.62) | 0.161 | 0.08 (-0.26 – 0.42) | 1.08 (0.77 – 1.52) | 0.655 |
| 2 | 0.12 (-0.24, 0.49) | 0.503 | 0.12 (-0.22 – 0.46) | 1.13 (0.80 – 1.58) | 0.491 |
| >2 | Ref |  | Ref |  |  |
| HCP suspected cancer |  |  |  |  |  |
| Yes | Ref |  | Ref |  |  |
| No | **1.400 (1.15, 1.644)** | **0.000** | **1.12 (0.77 – 1.48)** | **3.07 (2.15 – 4.39)** | **0.000** |
| Family size |  |  |  |  |  |
| <=2 | Ref |  | Ref |  |  |
| 2–5 | -0.007 (-0.26, 0.24) | 0.956 | -0.05 (-0.29 – 0.18) | 0.95 (0.75 – 1.20) | 0.657 |
| ≥6 | 0.23 (-0.146, 0.60) | 0.231 | -0.01 (-0.39 – 0.38) | 0.99 (0.68 – 1.46) | 0.975 |
| First health care facility contacts |  |  |  |  |  |
| Public hospital | Ref |  |  |  |  |
| Public health center | 0.290 (0.02, 0.55) | 0.030 | 0.07 (-0.19 – 0.32) | 1.07 (0.83 – 1.38) | 0.600 |
| Privet hospital/clinic | 0.165 (-0.16, 0.49) | 0.321 | 0.00 (-0.29 – 0.29) | 1.00 (0.75 – 1.34) | 0.990 |

*Note: HCP (health care provider), BE (breast examination)*

**Table 3:** Bivariate and Multivariable Loglogistic AFT Regression analysis of factors associated with longer time between diagnosis and initiation of treatment among women with breast cancer in Ethiopia

| Variable | Bivariate coefficient (95% CI) | P-value | | Multivariable coefficient (95% CI) | TR (95% CI) | P-value |
| --- | --- | --- | --- | --- | --- | --- |
| Age in year |  |  |  | |  |  |
| <40 | Ref |  |  | |  |  |
| 40–59 | -0.13 (-0.40, 0.12) | 0.305 | -0.13 (-0.382, 0.11) | | 0.87 (0.68, 1.12) | 0.280 |
| ≥60 | -0.26 (-0.65, 0.13) | 0.197 | -0.48 (-0.89, -0.07) | | **0.62 (0.41, 0.93)** | **0.021** |
| Residency |  |  |  | |  |  |
| Rural | -0.19(-0.48, 0.09) | 0.182 | -0.11 (-0.48, 0.26) | | 0.90 (0.62, 1.30) | 0.559 |
| Urban | Ref |  |  | |  |  |
| Study site |  |  |  | |  |  |
| Black Lion | -0.98 (-1.33, -0.62) | 0.000 | -0.46(-0.89, -0.02) | | **0.63 (0.41, 0.98)** | **0.038** |
| Jimma | -1.56 (-1.96, -1.17) | 0.000 | -1.33 (-1.83, -0.82) | | **0.26 (0.16, 0.44)** | **0.000** |
| Hiwot Fana | Ref |  | Ref | |  |  |
| Educational attainment |  |  |  | |  |  |
| No formal education | 0.02 (-0.30, 0.34) | 0.903 | 0.00 (-0.395, 0.39) | | 1.00 (0.67, 1.49) | 0.992 |
| Primary & Secondary | -0.009 (-0.31, 0.29) | 0.954 | -0.03 (-0.339, 0.26) | | 0.97 (0.71, 1.31) | 0.822 |
| Higher education | Ref |  |  | |  |  |
| Marital status |  |  |  | |  |  |
| Married | Ref |  |  | |  |  |
| Unmarried | 0.36(0.12, 0.61) | 0.003 | 0.37 (0.13, 0.62) | | **1.46 (1.14, 1.87)** | **0.002** |
| Occupational status |  |  |  | |  |  |
| Employed | Ref |  |  | |  |  |
| Unemployed | 0.14(-0.09, 0.37) | 0.244 | 0.16 (-0.09, 0.42) | | 1.18 (0.91, 1.53) | 0.218 |
| Monthly household income per capita (ETB) |  |  |  | |  |  |
| ≤2,100 (37 USD) | 0.11(-0.14, 0.38) | 0.383 | 0.38 (0.04, 0.73) | | **1.48 (1.05, 2.08)** | **0.026** |
| 2,101–3,254 (38-57 USD) | 0.35 (0.03, 0.67) | 0.029 | 0.25 (-0.02, 0.53) | | 1.29 (0.98, 1.70) | 0.075 |
| 3,255–15,000 (58-260 USD) | Ref |  |  | |  |  |
| Distance to the nearest health facility(km) |  |  |  | |  |  |
| <5 | Ref |  | Ref | |  |  |
| >=5 | -0.44 (-0.71, -0.16) | 0.002 | -0.20 (-0.47, 0.06) | | 0.82 (0.63, 1.06) | 0.132 |
| Health insurance |  |  |  | |  |  |
| Yes | Ref |  |  | |  |  |
| No | 0.18 (-0.11, 0.48) | 0.222 | 0.01 (-0.26, 0.29) | | 1.02 (0.77, 1.35) | 0.901 |
| Family history of breast cancer |  |  |  | |  |  |
| Yes | Ref |  |  | |  |  |
| No | 0.16 (-0.35, 0.68) | 0.542 | 0.34 (-0.11, 0.81) | | 1.41 (0.89, 2.25) | 0.144 |
| Traditional healing before diagnosis |  |  |  | |  |  |
| Yes | 0.63 (0.27, 0.99) | 0.001 | 0.29 (-0.03, 0.62) | | 1.34 (0.96, 1.87) | 0.084 |
| No | Ref |  |  | |  |  |
| Traditional healing after diagnosis |  |  |  | |  |  |
| Yes | 1.24 (0.69, 1.80) | 0.000 | **0.80 (0.28, 1.33)** | | **2.25 (1.33, 3.80)** | **0.003** |
| No | Ref |  |  | |  |  |
| Breast cancer literacy (0-5) |  |  |  | |  |  |
| 0 | 0.04 (-0.37, 0.45) | 0.844 | 0.05 (-0.38, 0.49) | | 1.06 (0.68, 1.64) | 0.804 |
| 1 | 0.18 (-0.23, 0.59) | 0.387 | 0.14 (-0.24, 0.52) | | 1.15 (0.78, 1.69) | 0.472 |
| 2 | -0.32 (-0.74, 0.09) | 0.130 | -0.22 (-0.61, 0.16) | | 0.80 (0.54, 1.18) | 0.259 |
| >2 | Ref |  |  | |  |  |
| Medically Confirmed chronic illness |  |  |  | |  |  |
| Yes | 0.08 (-0.21, 0.38) | 0.585 | 0.12 (-0.14, 0.40) | | 1.14 (0.86, 1.50) | 0.368 |
| No | Ref |  |  | |  |  |
| Painless breast mass as first symptom |  |  |  | |  |  |
| Yes | -0.43 (-0.83, -0.02) | 0.039 | -0.18 (-0.55, 0.19) | | 0.83 (0.57, 1.21) | 0.337 |
| No | Ref |  |  | |  |  |
| Stage of cancer |  |  |  | |  |  |
| Stage I & II | Ref |  | Ref | |  |  |
| Stage III or IV | 0.03(-0.20, 0.27) | 0.765 | 0.016 (-0.216, 0.249) | | 1.02 (0.81, 1.28) | 0.891 |
| Family size |  |  |  | |  |  |
| <2 | Ref |  | Ref | |  |  |
| 2-5 | -0.27 (-0.57, 0.02) | 0.066 | -0.02 (-0.30, 0.26) | | 0.98 (0.74, 1.30) | 0.883 |
| ≥6 | 0.113 (-0.31, 0.53) | 0.602 | 0.28 (-0.15, 0.72) | | 1.34 (0.86, 2.07) | 0.198 |
| Additional HCP consultations |  |  |  | |  |  |
| None | Ref |  | Ref | |  |  |
| One | -0.25 9-0.54, 0.07) | 0.125 | -0.12 (-0.42, 0.18) | | 0.89 (0.65, 1.20) | 0.431 |
| Two | -0.24 (-0.62, 0.13) | 0.204 | -0.18 (-0.54, 0.17) | | 0.83 (0.58, 1.19) | 0.312 |
| Three and more | -0.75 (-1.12, -0.38) | 0.000 | -0.08 (-0.61, 0.44) | | 0.92 (0.54, 1.56) | 0.750 |
| First HCP referral |  |  |  | |  |  |
| Yes | Ref |  | Ref | |  |  |
| No | 0.30 (0.02, 0.57) | 0.032 | 0.13 (-0.21, 0.49) | | 1.15 (0.81, 1.63) | 0.436 |
| HCP suspected cancer |  |  |  | |  |  |
| Yes | Ref |  | Ref | |  |  |
| No | 0.46 (0.17, 0.75) | 0.002 | 0.28 (-0.10, 0.66) | | 1.32 (0.90, 1.94) | 0.153 |
| First health care facility contacts |  |  |  | |  |  |
| Public hospital | Ref |  | Ref | |  |  |
| Public health center | 0.06 (-0.23, 0.35) | 0.677 | -0.15 (-0.44, 0.14) | | 0.86 (0.64, 1.16) | 0.317 |
| Privet hospital/clinic | -0.10 (-0.47, 0.26) | 0.578 | -0.04 (-0.38, 0.30) | | 0.96 (0.68, 1.35) | 0.814 |

**Table 4: Bivariate and Multivariable loglogistic AFT Regression analysis of factors associated with longer time between symptom detection and diagnosis among women with breast cancer in Ethiopia**

| Variable | Bivariate coefficient (95% CI) | P-value | Multivariable coefficient (95% CI) | TR (95% CI) | P-value |
| --- | --- | --- | --- | --- | --- |
| Age in years |  |  |  |  |  |
| <40 | Ref |  | Ref |  |  |
| 40–59 | 0.25 (0.00, 0.50) | 0.046 | 0.07 (-0.15, 0.30) | 1.08 (0.85, 1.36) | 0.528 |
| ≥60 | 0.48 (0.08, 0.88) | 0.018 | 0.16 (-0.23, 0.54) | 1.17 (0.80, 1.72) | 0.423 |
| Residency |  |  |  |  |  |
| Rural | 0.56 (0.30, 0.81) | 0.000 | 0.17 (-0.17, 0.50) | 1.18 (0.84, 1.66) | 0.336 |
| Urban | Ref |  |  |  |  |
| Educational attainment |  |  |  |  |  |
| No formal education | 0.77 (0.47, 1.07) | 0.000 | 0.33 (-0.04, 0.71) | 1.39 (0.96, 2.03) | 0.083 |
| Primary & Secondary Education | 0.06 (-0.24, 0.35) | 0.705 | -0.03 (-0.32, 0.27) | 0.97 (0.72, 1.31) | 0.854 |
| Higher education | Ref |  |  |  |  |
| Marital status |  |  |  |  |  |
| Married | Ref |  |  |  |  |
| Unmarried | 0.07 (-0.17, 0.31) | 0.570 | 0.07 (-0.16, 0.31) | 1.08 (0.85, 1.36) | 0.544 |
| Occupational status |  |  |  |  |  |
| Employed | Ref |  |  |  |  |
| Unemployed | 0.34 (0.11, 0.57) | 0.003 | -0.12 (-0.38, 0.13) | 0.88 (0.68, 1.14) | 0.339 |
| Distance to the nearest health facility(km) |  |  |  |  |  |
| Distance <5km | Ref |  |  |  |  |
| Distance ≥5km | 0.25 (-0.02, 0.51) | 0.075 | -0.05 (-0.30, 0.19) | 0.95 (0.74, 1.21) | 0.669 |
| Monthly household income per capita (ETB) |  |  |  |  |  |
| ≤2,100 (37 USD) | 0.58 (0.30, 0.87) | 0.000 | 0.10 (-0.23, 0.43) | 1.11 (0.80, 1.54) | 0.543 |
| 2,101–3,254 (38-57 USD) | 0.28 (-0.01, 0.58) | 0.060 | 0.12 (-0.16, 0.39) | 1.12 (0.85, 1.48) | 0.407 |
| 3,255–15,000 (58-260 USD) | Ref |  |  |  |  |
| Health insurance |  |  |  |  |  |
| Yes | Ref |  |  |  |  |
| No | -0.32 (-0.62, -0.03) | 0.033 | -0.19 (-0.45, 0.07) | 0.83 (0.64, 1.08) | 0.162 |
| Family history of breast cancer |  |  |  |  |  |
| Yes | Ref |  |  |  |  |
| No family history | 0.43 (-0.08, 0.94) | 0.101 | 0.02 (-0.41, 0.46) | 1.02 (0.66, 1.58) | 0.923 |
| Traditional healing before diagnosis |  |  |  |  |  |
| Yes | 0.89 (0.57, 1.20) | 0.000 | **0.76 (0.47, 1.05)** | **2.14 (1.60, 2.87)** | **0.000** |
| No | Ref |  |  |  |  |
| Family size |  |  |  |  |  |
| <2 | Ref |  |  |  |  |
| 2-5 | 0.13 (-0.17, 0.42) | 0.397 | 0.01 (-0.27, 0.28) | 1.00 (0.76, 1.32) | 0.973 |
| ≥6 | 0.32 (-0.07, 0.71) | 0.111 | 0.11 (-0.31, 0.53) | 1.12 (0.74, 1.69) | 0.603 |
| Breast cancer literacy (0-5) |  |  |  |  |  |
| 0 | 1.54 (1.13, 1.95) | 0.000 | **0.91 (0.47, 1.34)** | **2.47 (1.60, 3.81)** | **0.000** |
| 1 | 1.24 (0.83, 1.65) | 0.000 | **0.87 (0.48, 1.25)** | **2.38 (1.61, 3.51)** | **0.000** |
| 2 | 0.78 (0.36, 1.20) | 0.000 | **0.63 (0.23, 1.02)** | **1.87 (1.26, 2.78)** | **0.002** |
| >2 | Ref |  |  |  |  |
| Study site |  |  |  |  |  |
| Black Lion | 0.67 (0.31, 1.04) | 0.000 | **0.95 (0.56, 1.33)** | **2.58 (1.75, 3.79)** | **0.000** |
| Jimma | 0.71 (0.31, 1.11) | 0.000 | 0.41 (-0.04, 0.87) | 1.51 (0.96, 2.38) | 0.073 |
| Hiwot Fana | Ref |  |  |  |  |
| Painless breast mass as first symptom |  |  |  |  |  |
| Yes | 0.16 (-0.23, 0.55) | 0.416 | 0.13 (-0.21, 0.47) | 1.14 (0.81, 1.60) | 0.451 |
| No | Ref |  |  |  |  |
| Medically Confirmed chronic illness |  |  |  |  |  |
| Yes | 0.00 (-0.29, 0.30) | 0.996 | -0.01 (-0.27, 0.24) | 0.99 (0.77, 1.28) | 0.935 |
| No | Ref |  |  |  |  |
| First HCP referral |  |  |  |  |  |
| Yes | Ref |  |  |  |  |
| No | 0.29 (0.03, 0.54) | 0.028 | **0.39 (0.06, 0.71)** | **1.47 (1.06, 2.03)** | **0.019** |
| Additional HCP consultations |  |  |  |  |  |
| None | Ref |  |  |  |  |
| one | -0.16 (-0.47, 0.145) | 0.294 | -0.24 (-0.53, 0.04) | 0.78 (0.59, 1.04) | 0.094 |
| Two | -0.62 9-0.98, -0.262) | 0.001 | -0.22 (-0.56, 0.13) | 0.81 (0.57, 1.13) | 0.216 |
| Three or more | 0.29 (-0.05, 0.63) | 0.097 | **0.49 (0.02, 0.96)** | **1.63 (1.02, 2.62)** | **0.041** |
| HCP suspected cancer |  |  |  |  |  |
| Yes | Ref |  |  |  |  |
| No | 0.37 (0.09, 0.65) | 0.009 | 0.06 (-0.29, 0.41) | 1.06 (0.75, 1.50) | 0.748 |
|  |  |  |  |  |  |
| First health care facility contacts |  |  |  |  |  |
| Public health center | 0.54 (0.26, 0.83) | 0.000 | 0.26 (-0.02, 0.54) | 1.29 (0.98, 1.72) | 0.072 |
| Private hospital/clinic | -0.15 (-0.50, 0.21) | 0.411 | -0.04 (-0.37, 0.29) | 0.96 (0.69, 1.34) | 0.82 |
| Public Hospital | Ref |  |  |  |  |

**Table 5: Bivariate and multivariable Loglogistic AFT Regression analysis of factors associated with longer time between symptom detection and initiation of treatment among women with breast cancer in Ethiopia**

| Variable | Bivariate coefficient (95% CI) | P-value | Multivariable coefficient (95% CI) | TR (95% CI) | P-value |
| --- | --- | --- | --- | --- | --- |
| Age in years |  |  |  |  |  |
| <40 | Ref |  | Ref |  |  |
| 40–59 | 0.14 (-0.08, 0.36) | 0.201 | 0.00 (-0.20, 0.21) | 1.00 (0.82, 1.23) | 0.988 |
| ≥60 | 0.35 (-0.00, 0.69) | 0.051 | 0.06 (-0.29, 0.40) | 1.06 (0.75, 1.50) | 0.752 |
| Residency |  |  |  |  |  |
| Rural | 0.51 (0.29, 0.73) | 0.000 | 0.07 (-0.24, 0.37) | 1.07 (0.79, 1.46) | 0.664 |
| Urban | Ref |  |  |  |  |
| Educational attainment |  |  |  |  |  |
| No formal education | 0.70 (0.44, 0.97) | 0.000 | 0.19 (-0.15, 0.52) | 1.20 (0.86, 1.68) | 0.277 |
| Primary & Secondary Education | 0.15 (-0.10, 0.41) | 0.245 | -0.05 (-0.31, 0.21) | 0.95 (0.73, 1.23) | 0.724 |
| Higher education | Ref |  |  |  |  |
| Marital status |  |  |  |  |  |
| Married | Ref |  |  |  |  |
| Unmarried | 0.12 (-0.09, 0.33) | 0.27 | 0.13 (-0.08, 0.33) | 1.14 (0.92, 1.39) | 0.232 |
| Occupational status |  |  |  |  |  |
| Employed | Ref |  |  |  |  |
| Unemployed | 0.34 (0.14, 0.54) | 0.001 | -0.09 (-0.31, 0.14) | 0.92 (0.68, 1.14) | 0.455 |
| Distance to the nearest health facility(km) |  |  |  |  |  |
| Distance <5km | Ref |  |  |  |  |
| Distance ≥5km | 0.25 (0.00, 0.50) | 0.046 | -0.09 (-0.31, 0.13) | 0.91 (0.74, 1.21) | 0.425 |
| Monthly household income per capita (ETB) |  |  |  |  |  |
| ≤2,100 (37 USD) | 0.58 (0.30, 0.87) | 0.000 | 0.14 (-0.15, 0.43) | 1.15 (0.86, 1.54) | 0.348 |
| 2,101–3,254 (38-57 USD) | 0.28 (-0.01, 0.58) | 0.060 | 0.13 (-0.11, 0.37) | 1.14 (0.90, 1.45) | 0.283 |
| 3,255–15,000 (58-260 USD) | Ref |  |  |  |  |
| Health insurance |  |  |  |  |  |
| Yes | Ref |  |  |  |  |
| No | -0.32 (-0.62, -0.03) | 0.033 | -0.17 (-0.40, 0.06) | 0.84 (0.67, 1.08) | 0.148 |
| Family history of breast cancer |  |  |  |  |  |
| Yes | Ref |  |  |  |  |
| No family history | 0.43 (-0.08, 0.94) | 0.101 | 0.19 (-0.20, 0.59) | 1.21 (0.82, 1.80) | 0.334 |
| Traditional healing before diagnosis |  |  |  |  |  |
| Yes | **0.89 (0.57, 1.20)** | **0.000** | **0.61 (0.35, 0.87)** | **1.84 (1.42, 2.39)** | **0.000** |
| No | Ref |  |  |  |  |
| Family size |  |  |  |  |  |
| <2 | Ref |  |  |  |  |
| 2-5 | 0.13 (-0.17, 0.42) | 0.397 | 0.05 (-0.19, 0.29) | 1.05 (0.83, 1.33) | 0.672 |
| ≥6 | 0.32 (-0.07, 0.71) | 0.111 | 0.05 (-0.32, 0.42) | 1.05 (0.72, 1.52) | 0.798 |
| Breast cancer literacy (0-5) |  |  |  |  |  |
| 0 | 1.22 (0.87, 1.57) | 0.000 | **0.68 (0.30, 1.07)** | **1.98 (1.35, 2.89)** | **0.000** |
| 1 | 1.01 (0.66, 1.36) | 0.000 | **0.65 (0.30, 0.99)** | **1.91 (1.38, 2.45)** | **0.000** |
| 2 | 0.46 (0.09, 0.82) | 0.012 | **0.36 (0.01, 0.70)** | **1.43 (1.01, 2.06)** | **0.044** |
| >2 | Ref |  |  |  |  |
| Study site |  |  |  |  |  |
| Black Lion | **0.67 (0.31, 1.04)** | **0.000** | **0.46 (0.11, 0.81)** | **2.58 (1.74, 3.79)** | **0.011** |
| Jimma | 0.71 (0.31, 1.11) | 0.000 | 0.08 (-0.31, 0.47) | 1.51 (0.73, 2.38) | 0.697 |
| Hiwot Fana | Ref |  |  |  |  |
| Painless breast mass as first symptom |  |  |  |  |  |
| Yes | 0.16 (-0.23, 0.55) | 0.416 | 0.06 (-0.24, 0.36) | 1.06 (0.79, 1.45) | 0.693 |
| No | Ref |  |  |  |  |
| Medically Confirmed chronic illness |  |  |  |  |  |
| Yes | 0.00 (-0.29, 0.30) | 0.996 | 0.04 (-0.19, 0.27) | 1.04 (0.83, 1.31) | 0.736 |
| No | Ref |  |  |  |  |
| First HCP referral |  |  |  |  |  |
| Yes | Ref |  |  |  |  |
| No | 0.29 (0.03, 0.54) | 0.028 | **0.39 (0.10, 0.67)** | **1.47 (1.11, 1.95)** | **0.008** |
| Additional HCP consultations |  |  |  |  |  |
| None | Ref |  |  |  |  |
| one | -0.27 (-0.55, 0.00) | 0.045 | -0.26 (-0.51, -0.01) | 0.78 (0.60, 1.01) | 0.051 |
| Two and more | -0.54 (-0.82, -0.23) | 0.001 | -0.29 (-0.59, 0.01) | 0.75 (0.55, 1.01) | 0.059 |
| Three and more | 0.17 (-0.12, 0.47) | 0.252 | 0.28 (-0.13, 0.70) | 1.33 (0.88, 2.01) | 0.176 |
| HCP suspected cancer |  |  |  |  |  |
| Yes | Ref |  |  |  |  |
| No | 0.37 (0.09, 0.65) | 0.009 | 0.04 (-0.27, 0.35) | 1.05 (0.77, 1.42) | 0.78 |
| First health care facility contacts |  |  |  |  |  |
| Public health center | 0.54 (0.26, 0.83) | 0.000 | 0.14 (-0.11, 0.39) | 1.15 (0.90, 1.47) | 0.269 |
| Private hospital/clinic | -0.15 (-0.50, 0.21) | 0.411 | -0.11 (-0.40, 0.18) | 0.96 (0.67, 1.20) | 0.458 |
| Public Hospital | Ref |  |  |  |  |
| Traditional healing after diagnosis |  |  |  |  |  |
| Yes | 0.48 (0.08, 0.88) | 0.018 | -0.02 (-0.42, 0.39) | 0.98 (0.66, 1.47) | 0.934 |
| No | Ref |  |  |  |  |
| Stage of cancer |  |  |  |  |  |
| Stage I & II | Ref |  |  |  |  |
| Stage III or IV | -0.21 (-0.65, 0.24) | 0.36 | **0.29 (0.10, 0.49)** | **1.34 (1.11, 1.63)** | **0.003** |
